# When to consider intra-target microdosing: physiologically based pharmacokinetic modeling approach to quantitatively identify key factors for observing target engagement

**DOI:** 10.3389/fphar.2024.1366160

**Published:** 2024-07-25

**Authors:** Yasunori Aoki, Malcom Rowland, Yuichi Sugiyama

**Affiliations:** ^1^ Laboratory of Quantitative System Pharmacokinetics/Pharmacodynamics, Josai International University, Tokyo, Japan; ^2^ Drug Metabolism and Pharmacokinetics, Research and Early Development, Cardiovascular, Renal and Metabolism (CVRM), BioPharmaceuticals R&D, AstraZeneca, Gothenburg, Sweden; ^3^ Centre for Applied Pharmacokinetic Research, School of Pharmacy, University of Manchester, Manchester, United Kingdom; ^4^ iHuman Institute, ShanghaiTech University, Shanghai, China

**Keywords:** phase 0, PBPK, modeling and simulation, pharmacokinetics, target engagement, microdose study

## Abstract

Intra-Target Microdosing (ITM), integral to Phase 0 clinical studies, offers a novel approach in drug development, effectively bridging the gap between preclinical and clinical phases. This methodology is especially relevant in streamlining early drug development stages. Our research utilized a Physiologically Based Pharmacokinetic (PBPK) model and Monte Carlo simulations to examine factors influencing the effectiveness of ITM in achieving target engagement. The study revealed that ITM is capable of engaging targets at levels akin to systemically administered therapeutic doses for specific compounds. However, we also observed a notable decrease in the probability of success when the predicted therapeutic dose exceeds 10 mg. Additionally, our findings identified several critical factors affecting the success of ITM. These encompass both lower dissociation constants, higher systemic clearance and an optimum abundance of receptors in the target organ. Target tissues characterized by relatively low blood flow rates and high drug clearance capacities were deemed more conducive to successful ITM. These insights emphasize the necessity of taking into account each drug’s unique pharmacokinetic and pharmacodynamic properties, along with the physiological characteristics of the target tissue, in determining the suitability of ITM.

## Introduction

Microdosing, a component of Phase 0 studies, is a technique that can be used in the early stages of human drug development. On entering the systemic circulation this microdose is typically too small to cause any therapeutic effect or side effects, but is large enough to help characterize its metabolism and pharmacokinetics, using very highly sensitive analytical techniques (Sugiyama and Yamashita, 2011; Lappin et al., 2013; Burt et al., 2016; Burt et al., 2020). Microdoses can be administered and analyzed prior to Phase 1, potentially conserving valuable resources and facilitating decision making. According to Lappin et al. (Lappin et al., 2013), the linear scalability of microdoses to therapeutic doses has been observed in 27 out of 35 microdose trials, and is particularly attractive when alternative methods of preclinical prediction are considered problematic. The permitted dose of the human microdose is defined by regulation. It is: “Less than 1/100th of the dose (scaled on a mg/kg body weight basis) calculated to yield a pharmacological effect of the test substance based on primary pharmacodynamic data obtained from *in vitro* or *in vivo* non-clinical studies, with a maximum (adult) dose of up to 100 micrograms.” (International Conference on Harmonization, 2009)

Instead of giving a microdose of a new chemical entity systemically, Intra-Target Microdosing (ITM) involves administering a microdose directly into the specific area or “target” in the body where the drug is intended to act, via an incoming artery or direct administration into the target tissue (Burt et al., 2020). By this means it has the potential to generate local therapeutically active concentrations, which can be tracked using biomarkers. In general, ITM can be utilized to obtain very early evidence of in-human target engagement. As it implicitly takes advantage of selective distribution of a drug by means of route of administration ITM is not dependent *per se* on the drug or the target and, even in the case of a target being expressed in multiple tissues, so long as the particular tissue exposed to drug contains enough target to elicit a measurable response, this suffices.

We believe we can further extend the utility of ITM by focusing on a drug’s pharmacokinetic profile, together with receptor-drug kinetic data, to generate evidence to support the desirable systemic dose as part of human dose prediction. This early insight can be invaluable, by significantly curtailing the risks associated with subsequent stages, most notably the Phase II study. The alternative and traditional approach is to place significant reliance on the translational efficacy of drugs gained from preclinical species to human, which also carries significant inherent risks.

Intra-Target Microdosing (ITM), instead of giving a microdose of a new medicine systemically, involves administering a microdose directly to the specific area or “target” in the body where the drug is intended to act, via an incoming artery or direct administration into the target tissue (Burt et al., 2020). There have been various clinical studies using ITM focusing on comparing *in situ* drug effects of various compounds, particularly in oncology (Bhagavatula et al., 2021; Derry et al., 2023; Peruzzi et al., 2023). In general, ITM can be utilized to obtain evidence of in human target engagement of the investigating compound prior to phase 1 clinical trial.

Drug discovery and development are iterative processes. At the very outset, when a molecular target is identified, we already possess various independent physiologic knowledge, such as the approximate volume of the target tissue into which drug needs to distribute, as well as the relevant blood flow rate. Subsequent *in vitro* screenings offer insights into the feasible target affinity of various chemical series, as well as likely metabolic and transporter features. Once a candidate drug’s target profile is defined so can its approximate therapeutic dose, which subsequently informs the ITM dose. At each stage, modeling and simulation allows an estimate of the likelihood of observing the desired target engagement in human via ITM (defined as “ITM success”), so that it may guide the drug development team whether to consider including this step in the drug development plan.

By the time compounds are shortlisted for advancement, there’s an accumulation of important data: human therapeutic dose estimates, predicted *in vivo* PK data from preclinical species, human *in vitro* metabolic and transporter data, details on target tissue drug distribution, and correlations between exposure and target engagement biomarkers. With these data, constructing a PBPK-PD model akin to our simulation study becomes feasible, allowing for reasonably precise predictions regarding likely target engagement via ITM and establishing clear go-no-go decision benchmarks.

Our overarching goal is to strengthen the early stages of drug development by systematically evaluating the potential of ITM. More than just a technical tool, ITM can serve as an important decision-making criterion, optimizing investments and driving the evolution of drug development strategies. To support the approach, we have conducted a series of PBPK model-based Monte Carlo simulations of many virtual molecular entities, and compared their PK attributes against an array of marketed drugs, to assess the feasibility of observing acceptable target engagement through ITM trials. More specifically we aimed to answer the following two questions:

- Is there a possibility to observe target engagement through ITM, if so, what is the likely probability of achieving it?
- What kind of drugs have a higher chance of observing target engagement through ITM?

## Methods

### PBPK model-based Monte Carlo simulation

**FIGURE 1:**
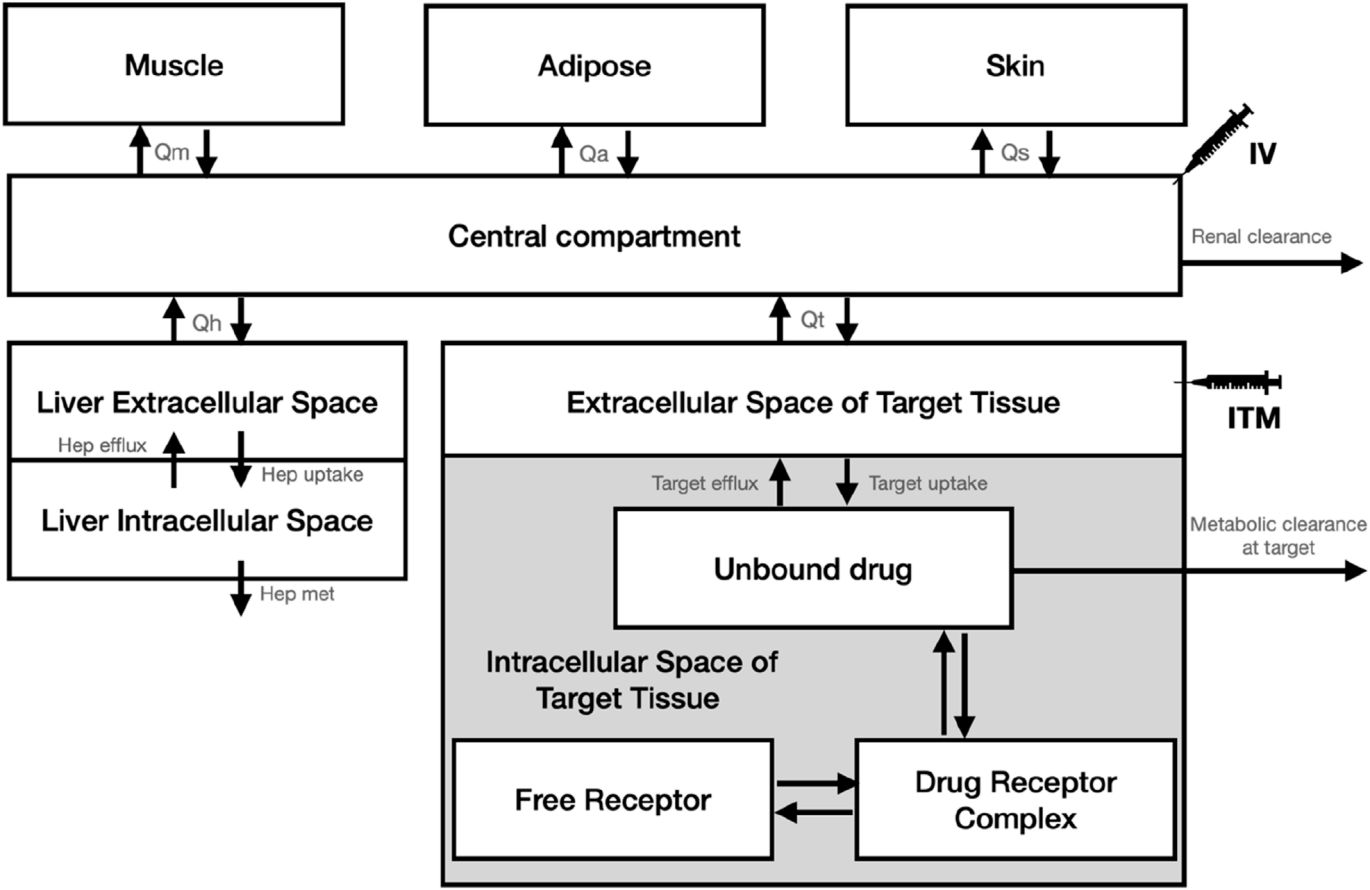
Schematic diagram of the PBPK model used for the Monte Carlo simulations.

We developed our analysis using a simplified PBPK model, tailored to assess pharmacokinetics and receptor occupancy (PK-RO), based on the model available in Koyama et al. (2021), schematically depicted in Figure 1. In our model, we maintained consistency by fixing all physiological parameters (tissue sizes and blood flows), except those directly related to the target, based on the values provided by Davies and Morris (Davies and Morris, 1993) and assuming all subjects in the trials weigh 75kg, as detailed in Table 1. For the physiological parameters of the target tissue, we opted for values utilized in the PBPK modeling of solid tumors, as documented by Rose et al. (Rose et al., 2019). The PBPK model, expressed as a system of ordinary differential equations, was numerically solved using the lsoda algorithm through its implementation in the rxode2 package (version 2.0.13) (Fidler et al., 2024) during Monte Carlo simulations in the R environment (R Core Team, 2023).

**TABLE 1:** Physiological parameters used for the PBPK model-based Monte Carlo simulations.

Symbol	Unit	Description	Value	Justification
Qa	L/hr	Blood flow rate to adipose	3.72*Weight*60/1000	Davies, B., and Morris (Davies and Morris, 1993)
Qh	L/hr	Blood flow rate to liver	20.7 *Weight*60/1000	
Qm	L/hr	Blood flow rate to muscle	10.7*Weight*60/1000	
Qs	L/hr	Blood flow rate to skin	4.28 *Weight*60/1000	
Va	L	Volume of skin	142 *Weight/1000	
Vh	L	Volume of liver	17.4 *Weight/1000	
Vhe	L	Volume of extracellular space of liver	6.69 *Weight/1000	
Vm	L	Volume of muscle	429 *Weight/1000	
Vs.	L	Volume of skin	111 *Weight/1000	
Vt	L	Volume of target	5/1000*(0.204 + 0.296)	Values that were used for Permeability-limited Tumor Model in (Rose et al., 2019)
Vt_ic	L	Volume of inner-cellular space of the target	5/1000*(1-0.204–0.296)	
Qt	L/hr	Blood flow rate to the target	0.686*60/1000*5	

Recognizing the necessity of differentiating between potential target tissues for ITM, we conducted a series of simulations across a range of tissue volumes and blood flow rates. In parallel, we simulated a variety of compounds, each designed to reflect the properties of existing small molecule drugs on the market.

To maintain simplicity and consistency across our experiments, we chose once-daily intravenous (IV) bolus administration as our primary route of therapeutic drug delivery. For the distribution of key kinetic parameters, we relied on the data from Kato et al. (Kato et al., 2003); for the distribution of target binding related parameters, we turned to the findings of Dahl and Akerud (Dahl and Akerud, 2013). We acknowledge that many pharmacokinetic parameters are heavily influenced by the specific chemical properties of a compound; therefore, we conducted simulations across a broad range of parameter distributions, ensuring that our calculations for intrinsic hepatic clearance (a measure of intracellular metabolic activity) closely mirrored the results presented by Kato et al. (Kato et al., 2003) The distribution of the parameters used for the simulation is specified in Table 2. All parameters followed a log-normal distribution, while some parameters with known correlations, as reported in Kato et al. (Kato et al., 2003), were simulated based on a multivariate log normal distribution. The physiological plausibility of the generated parameters is considered in the Results section of the manuscript.

**TABLE 2:** Kinetic parameters and their distributions used for PBPK model-based Monte Carlo simulation.

Parameter Name	Unit	Description	Distribution	Inter quartile range	Justification
Vmax_uptake	µmol/hr/body	Maximum uptake rate of active hepatic uptake	Log_normal	[10,1000]	Highly variable between compounds so conduct subset analyses in combination with other parameters related to liver uptake and metabolism
Km_uptake	µM	Michaelis-Menten constant for the hepatic uptake	Log_normal	[1/3,3]	
Vmax_met	µmol/hr/body	Maximum rate of hepatic metabolism	Log_normal	[25, 2500]	
Km_met	µM	Michaelis-Menten constant for the active hepatic metabolism	Log_normal	[10/3,30]	
PSdif_inf	L/hr/body	Diffusion coefficient for passive hepatic uptake	Log_normal	[3,300]	
Vmax_targetUptake	µmol/hr/body	Maximum rate of active uptake to the target organ	Log_normal	[0.0192, 1.92]	1/5 of hepatic metabolism normalized to the size of the organ
Km_targetUptake	µM	Michaelis-Menten constant for the active uptake to the target organ	Log_normal	[1/3,3]	
PSdiff_targetInflux = PSdiff_targetEflux	L/hr/body	Diffusion coefficient for passive uptake to the target organ. (assume passive influx and eflux are the same	Log_normal	[0.0115, 1.15]	Same as the passive uptake of the compound to liver normalized to the size of the organ
Vmax_targetMet	µmol/hr/body	Maximum metabolism rate for the inner target metabolism of the compound	Log_normal	[0.0192, 1.92]	1/5 of hepatic metabolism normalized to the size of the organ
Km_targetMet	µM	Michaelis-Menten constant for the inner target metabolism of the compound	Log_normal	[10/3,30]	Similar to km at liver
Kd	µmol/L	Equilibrium dissociation constant of the drug to the molecular target.	Log_normal	[0.0003, 0.006]	Dahl and Akerud (2013)
koff	1/hr	Dissociation constant of the drug to the molecular target.	Log_normal	[0.25, 4]	
X_TotalR	µmol	Total abundance of the receptor of the molecular target.	Log_normal	[0.001,0.1]	
Molar mass	g/mol	Molar mass	Fixed	400	
fp = fb		Plasma unbound fraction and in blood unbound fraction	Multivariable normal distribution (after log transformation) with mean and correlation matrix provided in Kato et al. (2003) We have used nPt/Kd to calculate fp and fb (note Kd here is the equilibrium dissociation constant to the plasma/blood protein and not to the molecular target) Kp_scaling is derived from the volume of distribution generated from this distribution		Kato et al. (2003)
CLr	L/hr/body	Renal clearance			
Kp_scaling		Scaling factor for the tissue partition coefficient			
Kpa		Partition coefficient for adipose	0.1* Kp_scaling		
Kpm		Partition coefficient for muscle	0.2*Kp_scaling		
Kps		Partition coefficient for skin	0.5*Kp_scaling		

To further probe the influence of these diverse parameters, and to bolster the robustness of our findings, we employed a sub-setting approach within our Monte Carlo simulations. We refer to compounds that made it through our Monte Carlo simulations, see criterion below, as “virtual compounds”.

### Assess the success of ITM for each virtual compound

We used fractional receptor occupancy (RO) as a quantitative measure of target engagement, and defined as “ITM success”, if the 24-h average RO (area under the RO curve/24 h) equals or exceeds 60%. Although RO can be measured using *in vivo* imaging methods such as PET imaging, more commonly, engagement is quantified with a biomarker, a rapidly responding, quantifiable, downstream measure of the drug-target interaction. The selection of the 60% benchmark is somewhat arbitrary, prompting us to explore the implications of this percentage on outcome by simulating different intended target occupancies. Though these conditions can be modified, and similar simulation strategies applied, the overarching insights drawn from the results remain largely unchanged. Therefore, to streamline our discussions, we consistently define “ITM success” as previously mentioned throughout this manuscript.

**FIGURE 2:**
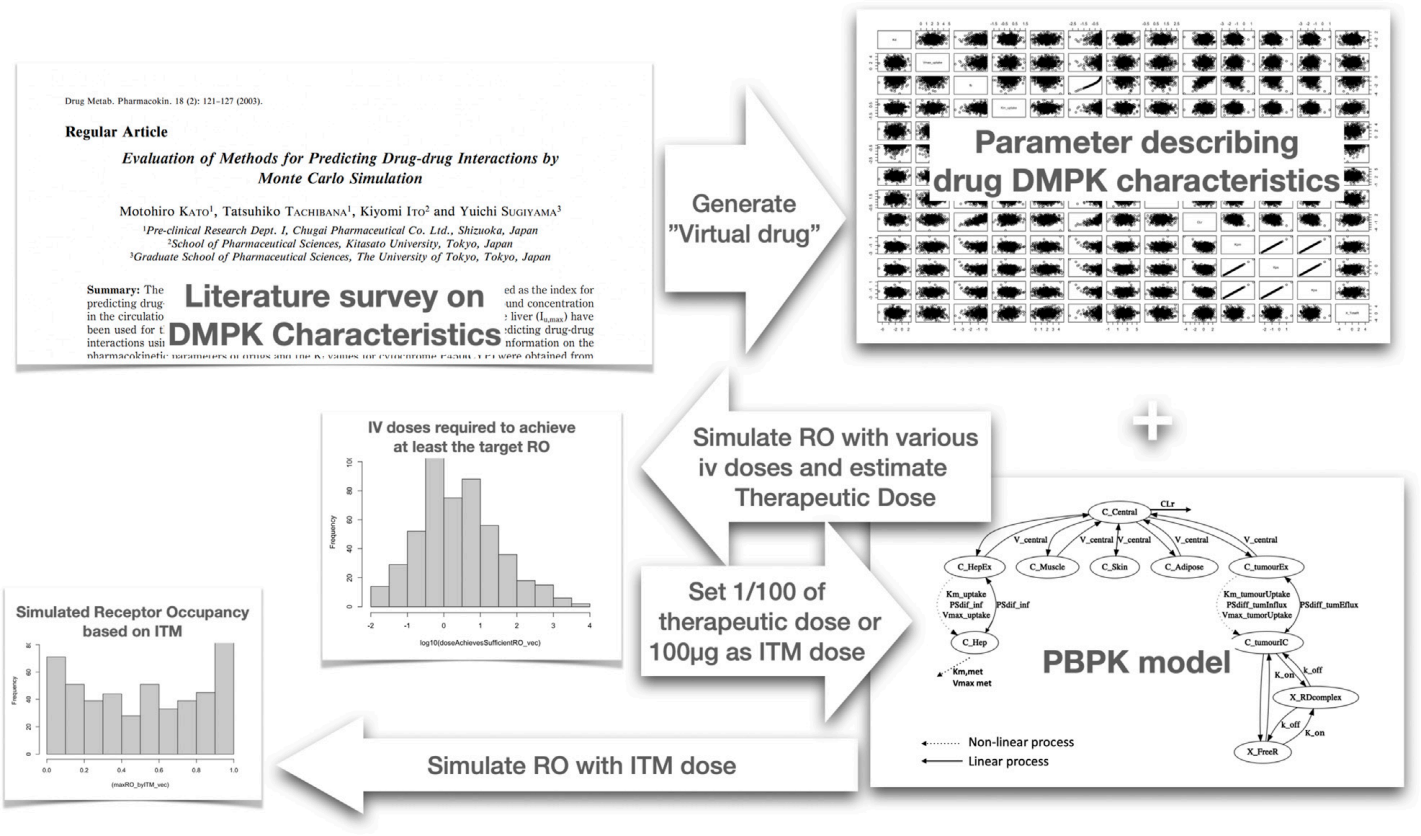
Schematic diagram of the Monte Carlo simulation we have conducted to determine probability of ITM success. We first analyzed literature data to identify the distribution of parameters for Monte Carlo simulation to create virtual compounds. Subsequently, for each virtual compound, we simulated the receptor occupancy (RO) across various intravenous (IV) doses using a physiologically based pharmacokinetic (PBPK) model. Based on the simulated RO, we selected the dose that achieves an average RO of above 60%, which we designated as the estimated therapeutic dose. Utilizing this estimated therapeutic dose calculate the microdose and then simulate the case of intra target administration using the same PBPK model to evaluate if the RO exceeds 60% on average. This process was repeated for every virtual compound generated, allowing us to calculate the probability of achieving successful ITM.

To practically gauge ITM success for each virtual compound, we executed the following procedure. A schematic diagram of the simulation study is depicted in Figure 2: Firstly, we simulated PK-RO across various IV doses to identify the dosage necessary to achieve the desired RO, terming this dosage as the “estimated therapeutic dose.” Next, a micro-dose was computed based on this “estimated therapeutic dose,” ensuring the micro-dose is the lesser of either 1/100th of the “estimated therapeutic dose” or 100µg, whichever is the lower. This was followed by running a PK-RO simulation where the micro-dose was delivered to the extracellular space or artery of the target tissue. (Here we make a key assumption that all the micro-dose is administered to the interstitial space of the target tissue.) The time-dependent RO profile was then analyzed to determine if it aligns with the target engagement objectives (i.e., more than 60% average RO).

### Summarize the success rate of the ITM

To understand the general trends concerning the probability of achieving the desired target engagement through ITM, we computed the probabilities of “ITM success”, as previously defined, across various Monte Carlo simulations.

As the main analysis, we produced 10,000 virtual compounds using the Monte Carlo simulation, with parameters distributed as specified in Tables 1, 2. This dataset allowed us to plot the correlation between the desired average RO and the probability of ITM success.

In our sensitivity analyses, we conducted Monte Carlo simulations with variations in three parameters explicitly included in the simulation model: blood flow rate to the target, equilibrium dissociation constant (Kd), and target receptor abundance. We performed these simulations by fixing one parameter at a time and varying it across seven fixed values. For computational efficiency, we generated 1,000 virtual compounds for each simulation, resulting in a total of 21,000 virtual compounds across all scenarios, and assessed ITM success for each of these 21 scenarios.

To extend the sensitivity analyses to parameters not explicitly present in the Monte Carlo simulations—such as the estimated therapeutic dose, hepatic clearance, and clearance at target organ—we conducted subgroup analyses. This involved dividing the Monte Carlo samples generated in the main analyses and constructing subgroups with a biased sample mean for the parameter of interest. Specifically, for each parameter, we divided the Monte Carlo simulation results into 10 bins by decile (i.e., 0%–10%, 10%–20%, 20%–30%, 30%–40%, 40%–50%, 50%–60%, 60%–70%, 70%–80%, 80%–90%, 90%–100% percentiles).

## Results

### Validation of the generated virtual compounds

**FIGURE 3:**
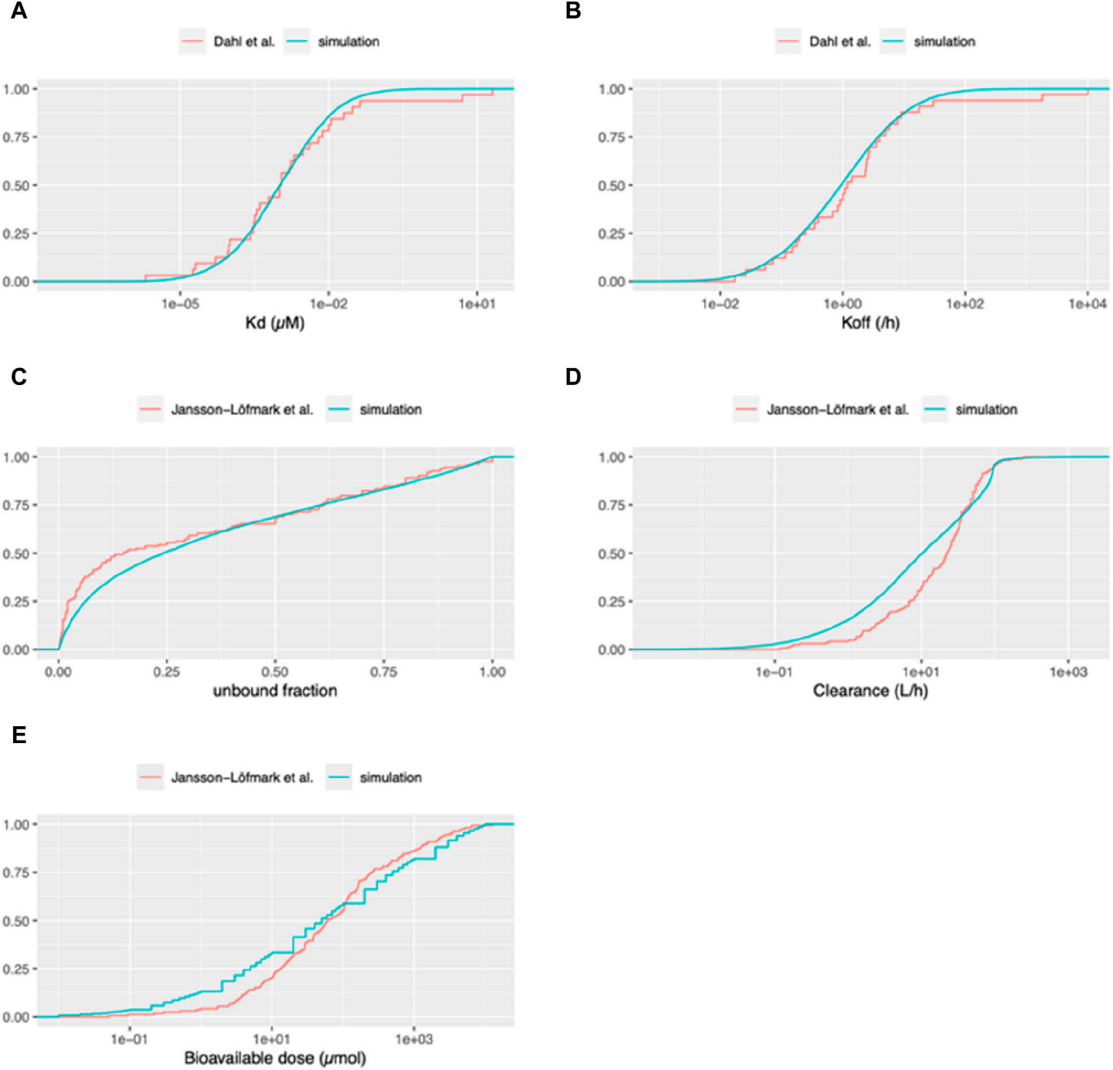
Cumulative distribution functions of pharmacokinetic parameters for virtual and marketed small molecule drugs. The blue curve depicts the distribution of parameters used for our Monte Carlo simulations. For panels **(A, B)**: The red curve represents the empirical cumulative distribution function (ECDF) based on the list of 33 compounds taken from Dahl and Akerud (2013). **(A)** Equilibrium dissociation constant of the drug-target receptor complex (Kd). **(B)** Dissociation rate constant of the drug-target receptor complex (koff). For panels **(C–E)**: The red curve represents the ECDF based on the list of 164 compounds from Jansson et al. (2020). **(C)** Unbound fraction in plasma (fu). **(D)** Total systemic clearance (sum of renal and hepatic clearance). **(E)** Bioavailable dose (calculated as bioavailability multiplied by daily dose for orally administered drugs).

In our initial analysis, we sought to validate the use of the log-normal distribution as a suitable approximation for the real parameter distribution. To achieve this, we generated values of Kd for the drug-target complex and the dissociation rate constant (koff) of the drug-target complex using a log-normal distribution, and subsequently compared these values with the actual distributions as presented by Dahl and Akerud, (Dahl and Akerud, 2013). As can be seen in Figures 3A, B, it is evident that the distributions of both parameters align closely with the experimentally observed log-normal distribution, thereby reinforcing its appropriateness as a representative model for these parameters.

We undertook an external validation by contrasting the compounds generated from our simulations with actual compound data from a publication that was not employed in creating our simulations. Utilizing the unbound fractions in plasma from Jansson et al. (2020), we compared these with those generated based on data from Kato et al. (2003). As illustrated in Figure 3C, our simulations produce less virtual compounds with unbound fractions lower than 0.5 compared to the data from Jansson et al. (2020).

Moreover, we assessed the distributions of clearance (combining renal clearance and intrinsic hepatic clearance) between our simulated compounds and those currently available as medicines on the market, as per Jansson-Löfmark et al. (Jansson et al., 2020) data. As can be seen in Figure 3D, although it is not perfect, clearance distribution of our simulated compounds aligns reasonably well with the data presented in Jansson et al. (2020). To further validate our methodology for estimating the therapeutic dose, we compared the distribution of the daily bioavailable doses from our simulated compounds against the data in Jansson et al. (2020). In this study, we compared the bioavailable doses, given that the data from actual small molecule compounds included a mix of oral and IV administration. Since our simulation consists only of IV doses, we standardized the dose for those drugs intended for oral administration by adjusting the daily dose according to their bioavailabilities, thus reflecting the amount that enters the systemic circulation. As can be seen in Figure 3E, the cumulative density of the “estimated therapeutic dose” and actual doses of the drugs on the market, while exhibiting certain deviations, follow a similar trend across the range of doses. Specifically, for lower bioavailable doses (around 0.1–10 micromoles, corresponding to 0.04–4 mg for a 400 MW compound), the simulated dataset tends to report a slightly higher fraction of compounds compared to the dataset of Jansson et al. (2020). As the dose increases, both curves are closely aligned, especially around the middle dose range. Toward the higher end of the dose spectrum, there is a slight divergence again, with the simulation curve being slightly below the Jansson et al. (2020) curve. Additionally, in the dataset generated by Jansson et al. (2020), the therapeutic dose for 35 out of 164 drugs (approximately 21%) was less than 10 mg daily. This implies that for about 80% of the drugs, the microdose was set at 100 µg, regardless of the therapeutic dose.

### Estimation of therapeutic dose, simulation of time-course of drug concentrations, RO and success probability of ITM

Our methodology begins by determining the therapeutic dose for IV administration, using RO as an efficacy indicator. An IV dose is deemed as an estimated therapeutic dose if it sustains an average RO above 60% for 24 h. Based on this, a microdose, defined as 1% of the effective dose and capped at 100 µg, is administered directly to the artery of the target organ to evaluate efficacy maintenance at the microdose level through simulation studies. This approach is detailed in Figure 2. Monte Carlo simulations were conducted, and the summary statistics of generated parameters were listed in Supplementary Table S1. From these, nine compounds were selected to exemplify the procedure. The results, documented in Supplementary Figure S1-9, illustrate the RO change over time with different IV doses to ascertain the effective IV dose, and RO over time for both the estimated therapeutic dose via IV and ITM doses. Supplementary Table S2 details the primary biochemical parameters for these nine compounds. Among them, two compounds had therapeutic doses exceeding 25 µmol (10 mg), with a microdose limit of 0.25 µmol (100 µg). The RO at ITM varied widely, ranging from 6% to 93%, and three compounds achieved ITM success (RO>60%). The reasons for this variation can be understood by examining the biochemical parameters (especially total clearance CLh + CLr, receptor abundance and Kd), of each compound, as shown in Supplementary Table S2.

### Simulated success rates of the ITM

#### Influence of the target organ

**FIGURE 4:**
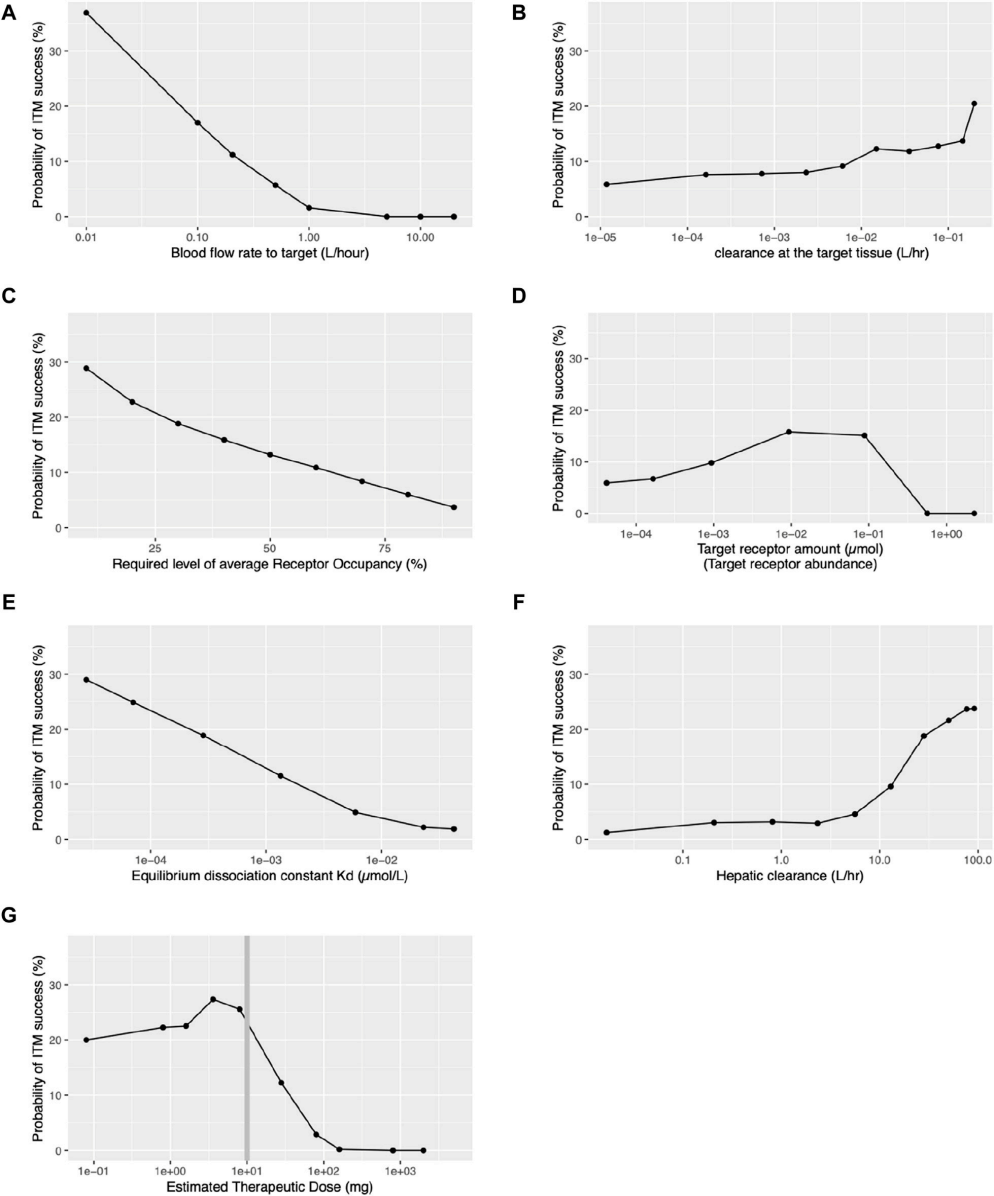
Relationships between probability of ITM success and various key target organ, target receptor, and pharmacokinetic parameters. ITM success is defined as achieving an average receptor occupancy of over 60% during the 24-h period following ITM administration, except for panel **(C)**. **(A)** Blood flow rate to the target tissue. **(B)** Clearance within the target tissue. **(C)** Required level of receptor occupancy. **(D)** Target receptor abundance. **(E)** Equilibrium dissociation constant (Kd) for the drug-target receptor complex. **(F)** Hepatic clearance. **(G)** Estimated therapeutic dose.

In our investigation, we identified that two factors related to the target organ - blood flow rate to the target organ and clearance at the target organ - influence the probability of ITM success. Figure 4A illustrates the quantitative relationship between the probability of ITM success and the blood flow rate to the target. It suggests that a smaller blood flow rate to the target correlates with a higher probability of ITM success. Figure 4B demonstrates how the clearance capacity of the target organ influences the probability of ITM success. A higher clearance rate at the target tissue corresponds to a higher probability of ITM success. However, even with minimal target organ clearance, the probability of ITM success remains above 5%, suggesting that even if there is no clearance at the target tissue, one can still achieve successful target engagement via ITM.

#### Influence of the target receptor

Our investigation has revealed that the following target receptor-related factors influence the probability of ITM success: the required level of average RO, the target receptor abundance, and Kd for the drug-target complex. The desired level of average RO significantly influences the probability of ITM success. As illustrated in Figures 4A,C decrease in the desired level of average RO leads to an increase in the probability of ITM success. Figure 4D displays the relationship between ITM success probability and target receptor abundance, showing a moderate increase up to 0.001–0.1µmol, followed by a steep decrease. In contrast, Figure 4E presents the monotone relationship between Kd and the probability of ITM success, with results indicating that a smaller Kd results in a higher probability of ITM success.

#### Influence of systemic pharmacokinetics factors

Although it can be expected that the nature of the target organ and receptor significantly influences the ITM success rate, the drug concentration at the target organ also plays a crucial role. Our investigation has revealed that hepatic clearance and estimated therapeutic dose, both systemic pharmacokinetics-related factors, influence the probability of ITM success. As clearly demonstrated in Figure 4F, the hepatic clearance of a compound significantly impacts the probability of ITM success, with an increasing trend observed as hepatic clearance rises beyond 10 L/h. However, when hepatic clearance is less than 1L/hr, the probability of ITM success falls below 3%. We also found that the estimated therapeutic dose significantly affects the probability of ITM success. Figure 4G portrays the relationship between the estimated therapeutic dose and the probability of ITM success. Remarkably, success probability surpasses 20% when the estimated therapeutic dose is below 10mg, but sharply declines for doses above this level. It is crucial to note that for doses under 10mg, the microdose is 1/100th of the therapeutic dose, while for doses exceeding 10mg, the microdose remains a constant 100 µg, regardless of the therapeutic dose. It should be noted that all the qualitative relationships remained consistent across all levels of required receptor occupancy, as presented in Supplementary Figure S10. Additionally, the distributions of the receptor occupancy are depicted in Supplementary Figure S11.

## Discussion

ITM offers an innovative approach to drug development, potentially augmenting the transition from preclinical to clinical. Our study provides an in-depth analysis of various factors influencing the probability of successful target engagement via ITM. To investigate this, we conducted PBPK model-based Monte Carlo studies. Despite the many simplifying assumptions made to make this study feasible, both our internal and external validations confirm the overall reasonableness of these assumptions (cf. Figure 3). This ensured that the distribution of the key compound characteristics of the Monte Carlo-generated “virtual compounds” are similar to what is on the market, i.e., approved medicines.

Primarily, our PBPK model-based simulation indicated that for certain compounds, achieving target engagement by ITM at RO levels comparable to therapeutic doses is feasible. While the likelihood is largely dictated by the target tissue and compound properties, our simulations revealed that if the estimated therapeutic dose is under 10mg, approximately 20% of compounds should exhibit successful target engagement via ITM (Figure 4G). Nonetheless, the potential utility of ITM must be evaluated at each phase of drug discovery, considering both the target tissue and compound characteristics.

One observation was the inverse relationship observed between the blood flow rate to the target organ and the probability of successful target engagement (cf. Figure 4A), the reason being that any increase in flow rate will lower the likelihood of retention of the administered microdose at the target site. Hence, the chances of successful ITM are improved when blood flow to the target organ is either inherently low or physically restricted, compatible with physiological constraints.

We also have demonstrated that the required level of receptor occupancy heavily influences the probability of ITM success, that is to say, the lower the required level of receptor occupancy level to observe target engagement, the higher the chance of ITM success (cf. Figure 4C). As the required receptor occupancy level decreases, the estimated therapeutic dose will also decrease, resulting in fewer compounds being subject to the restriction of the 100 µg limit of the microdose, thus causing a higher ITM success rate. It is typically understood that agonists necessitate lower target occupancy compared to antagonists. Hence, ITM may be more suitable when developing agonists. Although this may seem trivial, a decrease in the required level of receptor occupancy leads to a lower estimated therapeutic dose and, consequently, the ITM dose. Therefore, the balance between the required RO and the probability of ITM success is not necessarily trivial.

While the Kd pertinent to potency is refined until the late phases of preclinical drug discovery, the probable range of this constant for a particular chemical series is often discernible during *in vitro* compound screening. Hence, we probed the interrelation between Kd and ITM success probability and found that the smaller Kd higher the probability of ITM success (cf, Figure 4E). This suggests that recent progress in the chemistry optimization towards single nano molar range potency will potentially increase the potential utility of ITM.

Hepatic clearance is a pivotal metric influencing pharmacokinetics. When the drug is administered via IV route, then the hepatic clearance does influence the drug exposure at the target tissue. For the high clearance drug, it requires more drug to achieve the same level of target exposure compared to the low clearance drug. In other words, the high clearance drugs have higher estimated therapeutic dose. When the estimated therapeutic dose increases then the micro-dose increases. On the other hand, when the drug is administered to the target tissue, the influence of hepatic clearance to the average receptor occupancy is rather limited. Thus, for the high clearance drug more drug will be delivered to the target via ITM hence ITM success will increase.

Figure 4F delineates the influence of hepatic clearance and ITM success probability. There’s a marked increase in success probability as hepatic clearance increases, especially evident when exceeding 10 L/h. Generally, there is a tendency as part of early drug development to reduce hepatic clearance, as this leads to longer half-lives and reduced daily dose requirements. This in turn reduces the probability of ITM success. However, as illustrated in Figure 3E Jansson-Löfmark et al.‘s survey (Jansson et al., 2020) show that more than 75% of marketed small molecules have a larger than 10 L/h total body clearance. Thus, even with the progressive effort to design molecules with lower hepatic clearance, it is unlikely to reduce sufficiently to generally make ITM infeasible.

We have also observed that clearance at the target tissue plays a modest role in the success of ITM as indicated in Figure 4B. An increase in clearance within the target organ (CL_target) can enhance the probability of ITM success. This is because, even though CL_target is elevated, it does not significantly impact the total body clearance. A higher CL_target improves the extraction ratio across the target tissue, necessitating an increased the estimated therapeutic dose. Consequently, the amount of the drug reaching the target tissue escalates with the higher estimated therapeutic dose, thereby increasing the likelihood of achieving the desired RO.

Lastly, at the end of preclinical studies, it should be noted that when the predicted daily therapeutic dose exceeds 10 mg there is a projected very low probability of ITM success (cf. Figure 4G). This hinges on the current definition of a microdose which cannot exceed 100 µg or 1/100th of the estimated therapeutic dose. This also explains a similar bi-phasic relationship between the probability of ITM success and the target receptor abundance. As the receptor abundance increases so does the estimated therapeutic dose (cf. Supplementary Figure S12) which leads to the increase in the ITM dose hence the ITM success. On the other hand, with the ITM dose capped at 100µg, it is impossible to achieve ITM success when the receptor abundance is more than the mol amount of the microdose (0.25µmol, assuming a molecular weight of 400 g/mol).

While our simulations indicated that the virtual compounds generated exhibit similar distribution characteristics to marketed small molecule drugs, it is important to recognize that marketed compounds represent a very small fraction of those considered for drug products. Therefore, the distribution of our virtual compounds might not accurately reflect the broader spectrum of compounds currently under consideration for ITM. Additionally, our simulation relies on the definition of a microdose as per current guidelines (International Conference on Harmonization, 2009), which assumes no therapeutic effect or side effects when administered systemically or orally. However, our simulation suggests that altering the administration location could potentially lead to target engagement. This observation necessitates further discussion and reconsideration of the microdose definition for ITM, ensuring both volunteer safety and the informativeness of such trials.

It should be emphasized that the success rate of ITM demonstrated in this work is based on virtual compounds generated via Monte Carlo simulations, which mimic the distribution of the properties of drugs on the market. This reflects the probability of observing target engagement via ITM on a compound randomly selected from those currently on the market. In reality, before making an investment decision for ITM, there should be sufficient information from preclinical experiments to determine the probability of observing target engagement via ITM, as demonstrated in this paper. Only compounds with a sufficient probability of ITM success should be considered for ITM. Thus, the probability of observing target engagement via ITM should be significantly higher for a compound chosen for an ITM study.

In our modeling approach, we recognize several key limitations that warrant mention. First, we have simplified the analysis by assuming that active uptake into the target tissue is proportional to hepatic uptake and scales with the volume of the target tissue, due to the absence of specific uptake data. Also, the PBPK model used throughout the study may not fully capture the complexities of *in vivo* pharmacokinetics. Furthermore, our simulation does not take account of variability in the relationship between target receptor occupancy and the measurable target engagement of biomarkers. Instead, we have presumed direct observability of receptor occupancy. This assumption could limit the model’s applicability to molecular targets that lack sensitive circulating target engagement biomarkers. Such simplification, while necessary for the feasibility of our study, is an acknowledged limitation that could affect the extrapolation of our results to a “real-world” setting.

It is important to note that the decision to further narrow down candidate compounds through ITM clinical trials or to proceed directly to Phase-1 trials based solely on pre-clinical predictive results rests with the comprehensive judgment of each study sponsor. However, it is worth mentioning that according to statistics from 2005 to 2015, the probability of compounds that advanced to Phase-1 trials through traditional selection methods eventually being approved and entering the market ranges from only five rising to 14% (Wong et al., 2019). This highlights the undeniable need for new methodologies in the selection process of compounds.

## Conclusion

ITM is a promising method in drug development, yet its implementation needs thorough evaluation. This involves considering not only the physiological characteristics of the target tissue but also each drug’s distinct pharmacokinetic and receptor binding properties, along with practical considerations. Our PBPK model-based simulations suggest that ITM could achieve adequate target engagement for certain compounds. We recommend ITM particularly for cases where the target tissue has limited blood flow rate, and the anticipated therapeutic dose is below 10 mg. This is to ensure that the 1% of therapeutic dose does not exceed 100 µg. For therapeutic doses under 10mg, our estimates indicate that over 20% of compounds might show effective target engagement using ITM. Additionally, it is crucial for these compounds to demonstrate strong potency, characterized by Kd in the nanomolar range, and to have a reasonable rate of hepatic clearance. These criteria help in maximizing the potential success of ITM in drug development.

## Data Availability

The raw data supporting the conclusion of this article will be made available by the authors, without undue reservation.

## References

[B1] Bhagavatula S. Thompson D. Ahn S. W. Upadhyaya K. Lammers A. Deans K. (2021). A miniaturized platform for multiplexed drug response imaging in live tumors. Cancers 13 (4), 653. 10.3390/cancers13040653 33562152 PMC7915324

[B2] Burt T. Yoshida K. Lappin G. Vuong L. John C. de Wildt S. N. (2016). Microdosing and other phase 0 clinical trials: facilitating translation in drug development. Clin. Transl. Sci. 9 (2), 74–88. 10.1111/cts.12390 26918865 PMC5351314

[B3] Burt T. Young G. Lee W. Kusuhara H. Langer O. Rowland M. (2020). Phase 0/microdosing approaches: time for mainstream application in drug development? Nat. Rev. Drug Discov. 19 (11), 801–818. 10.1038/s41573-020-0080-x 32901140

[B4] Dahl G. Akerud T. (2013). Pharmacokinetics and the drug–target residence time concept. Drug Discov. today 18 (15-16), 697–707. 10.1016/j.drudis.2013.02.010 23500610

[B5] Davies B. Morris T. (1993). Physiological parameters in laboratory animals and humans. Pharm. Res. 10 (7), 1093–1095. 10.1023/a:1018943613122 8378254

[B6] Derry J. Burns C. Frazier J. P. Beirne E. Grenley M. DuFort C. C. (2023). Trackable intratumor microdosing and spatial profiling provide early insights into activity of investigational agents in the intact tumor microenvironment. Clin. Cancer Res. 29 (18), 3813–3825. 10.1158/1078-0432.CCR-23-0827 37389981 PMC10502463

[B7] Fidler M. Hallow M. Wilkins J. Wang W. (2024). RxODE: facilities for simulating from ODE-based models. R package version 2.1.2. Available at: https://CRAN.R-project.org/package=RxODE.

[B8] International Conference on Harmonization (2009). “Guidance on nonclinical safety studies for the conduct of human clinical trials and marketing authorization for pharmaceuticals M3 (R2),” in International conference on harmonisation of technical requirements for registration of pharmaceuticals for human use (Geneva, Switzerland: ICH Secretariat).

[B9] Jansson L. R. Hjorth S. Gabrielsson J. (2020). Does *in vitro* potency predict clinically efficacious concentrations? Clin. Pharmacol. Ther. 108 (2), 298–305. 10.1002/cpt.1846 32275768 PMC7484912

[B10] Kato M. Tachibana T. Ito K. Sugiyama Y. (2003). Evaluation of methods for predicting drug-drug interactions by Monte Carlo simulation. Drug Metabolism Pharmacokinet. 18 (2), 121–127. 10.2133/dmpk.18.121 15618726

[B11] Koyama S. Toshimoto K. Lee W. Aoki Y. Sugiyama Y. (2021). Revisiting nonlinear Bosentan pharmacokinetics by physiologically based pharmacokinetic modeling: target binding, albeit not a major contributor to nonlinearity, can offer prediction of target occupancy. Drug Metabolism Dispos. 49 (4), 298–304. 10.1124/dmd.120.000023 33558262

[B12] Lappin G. Noveck R. Burt T. (2013). Microdosing and drug development: past, present and future. Expert Opin. drug metabolism Toxicol. 9 (7), 817–834. 10.1517/17425255.2013.786042 PMC4532546 23550938

[B13] Peruzzi P. Dominas C. Fell G. Bernstock J. D. Blitz S. Mazzetti D. (2023). Intratumoral drug-releasing microdevices allow *in situ* high-throughput pharmaco phenotyping in patients with gliomas. Sci. Transl. Med. 15 (712), eadi0069. 10.1126/scitranslmed.adi0069 37672566 PMC10754230

[B14] R Core Team (2023) R: a language and environment for statistical computing_. Vienna, Austria: R Foundation for Statistical Computing.

[B15] Rose R. H. Sun K. Li L. Gada K. Wang J. Y. Qiu Y. (2019). Abstract 2952: predicting concentration of PARP inhibitors in human tumor tissue using PBPK modeling. Cancer Res. 79 (13), 2952. 10.1158/1538-7445.am2019-2952

[B16] Rowland M. McLachlan A. (1996). Pharmacokinetic considerations of regional administration and drug targeting: influence of site of input in target tissue and flux of binding protein. J. Pharmacokinet. Pharmacodynamics 24, 369–387. 10.1007/BF02353518 9044166

[B17] Sugiyama Y. Yamashita S. (2011). Impact of microdosing clinical study—why necessary and how useful? Adv. drug Deliv. Rev. 63 (7), 494–502. 10.1016/j.addr.2010.09.010 20950660

[B18] Van Nuland M. Rosing H. Thijssen B. Burgers J. A. Huitema A. D. R. Marchetti S. (2020). Pilot study to predict pharmacokinetics of a therapeutic gemcitabine dose from a microdose. Clin. Pharmacol. Drug Dev. 9 (8), 929–937. 10.1002/cpdd.774 31970932

[B19] Wong C. H. Siah K. W. Lo A. W. (2019). Estimation of clinical trial success rates and related parameters. Biostatistics 20 (2), 273–286. 10.1093/biostatistics/kxx069 29394327 PMC6409418

