# Supplementary material for: When to consider intra-target microdosing: physiologically based pharmacokinetic modeling approach to quantitatively identify key factors for observing target engagement
Source: Front Pharmacol. 2024 Jul 25;15:1366160. doi: 10.3389/fphar.2024.1366160 (PMC11306728; doi:10.3389/fphar.2024.1366160)
Supplement: Supplementary file 2 [file DataSheet2.docx]

When to consider Intra-Target Microdosing: Physiologically Based Pharmacokinetic Modeling Approach to Quantitatively Identify Key Factors for Observing Target Engagement

Yasunori Aoki^1,2^, Malcom Rowland^3^, and Yuichi Sugiyama^1^

^1^ Laboratory of Quantitative System Pharmacokinetics/Pharmacodynamics, Josai International University, Tokyo, Japan

^2^ Drug Metabolism and Pharmacokinetics, Research and Early Development, Cardiovascular, Renal and Metabolism (CVRM), BioPharmaceuticals R&D, AstraZeneca, Gothenburg, Sweden

^3^ Centre for Applied Pharmacokinetic Research, School of Pharmacy, University of Manchester, Manchester, U.K.

# Supplementary Material

Supplementary Table 1: Summary statistics of the parameter distribution used for the main study.

| **Variable** | **N** | **Mean** | **Std. Dev.** | **Min** | **Pctl. 25** | **Pctl. 50** | **Pctl. 75** | **Max** |
| --- | --- | --- | --- | --- | --- | --- | --- | --- |
| Weight (kg) | 10000 | 75 | 0 | 75 | 75 | 75 | 75 | 75 |
| Kd (µmol/L) | 10000 | 0.017 | 0.14 | 0.000000035 | 0.00031 | 0.0014 | 0.006 | 7.6 |
| k_on (1/M hr) | 10000 | 72995 | 1614723 | 0.0095 | 99 | 739 | 5588 | 130289791 |
| Vmax_uptake (µmol/hr/body) | 10000 | 21482 | 362365 | 0.000024 | 9.5 | 103 | 1061 | 26560432 |
| fb | 10000 | 0.36 | 0.32 | 0.00006 | 0.071 | 0.26 | 0.62 | 1 |
| Km_uptake (µM) | 10000 | 3.6 | 9.9 | 0.0016 | 0.33 | 0.97 | 2.9 | 230 |
| PSdif_inf (L/hr/head) | 10000 | 18061 | 841961 | 0.000099 | 3 | 30 | 296 | 80515657 |
| fh | 10000 | 0.71 | 0.29 | 0.00079 | 0.5 | 0.83 | 0.96 | 1 |
| Vmax_met (µmol/hr/body) | 10000 | 78194 | 2317129 | 0.0016 | 24 | 259 | 2494 | 153767002 |
| Km_met (µM) | 10000 | 38 | 109 | 0.032 | 3.4 | 10 | 30 | 3237 |
| Vmax_tumorUptake (µmol/hr/body) | 10000 | 35 | 522 | 0.00000062 | 0.02 | 0.2 | 2 | 37443 |
| Km_tumourUptake (µM) | 10000 | 3.6 | 10 | 0.002 | 0.33 | 1 | 3 | 230 |
| PSdiff_tumInflux (L/hr/head) | 10000 | 35 | 833 | 0.00000006 | 0.011 | 0.12 | 1.2 | 64341 |
| PSdiff_tumEflux (L/hr/head) | 10000 | 35 | 833 | 0.00000006 | 0.011 | 0.12 | 1.2 | 64341 |
| ft | 10000 | 0.71 | 0.29 | 0.00079 | 0.5 | 0.83 | 0.96 | 1 |
| V_central (L) | 10000 | 6 | 0 | 6 | 6 | 6 | 6 | 6 |
| CLr (L/hr/body) | 10000 | 12 | 48 | 0.000041 | 0.36 | 1.7 | 6.7 | 1762 |
| Kpm | 10000 | 0.14 | 0.31 | 0.00028 | 0.022 | 0.052 | 0.13 | 7.8 |
| Kps | 10000 | 0.35 | 0.77 | 0.00071 | 0.054 | 0.13 | 0.33 | 20 |
| Kpa | 10000 | 0.07 | 0.15 | 0.00014 | 0.011 | 0.026 | 0.067 | 3.9 |
| Km_tumMet (µM) | 10000 | 37 | 122 | 0.038 | 3.4 | 10 | 31 | 4642 |
| Vmax_tumMet (µmol/hr/body) | 10000 | 72 | 1820 | 0.00000054 | 0.018 | 0.19 | 1.9 | 102211 |
| X_TotalR (µmol) | 10000 | 2.5 | 49 | 0.000000006 | 0.00093 | 0.01 | 0.094 | 3477 |
| nPt_div_Kd | 10000 | 34 | 275 | 0.00032 | 0.62 | 2.8 | 13 | 16727 |
| CLr_div_fp (L/hr/body) | 10000 | 33 | 117 | 0.0089 | 2.7 | 8.5 | 26 | 6530 |
| V_central (L) | 10000 | 6 | 0 | 6 | 6 | 6 | 6 | 6 |
| Qt (L/hr) | 10000 | 0.21 | 0 | 0.21 | 0.21 | 0.21 | 0.21 | 0.21 |
| CLinflux (L/hr/body) | 10000 | 136822 | 5652136 | 0.03 | 67 | 479 | 3641 | 544769922 |
| Clint_m (L/hr/body) | 10000 | 32643 | 1962743 | 0.000042 | 2 | 24 | 291 | 193503417 |
| PSeff (L/hr/body) | 10000 | 18061 | 841961 | 0.000099 | 3 | 30 | 296 | 80515657 |
| Clint_all (L/hr/body) | 10000 | 39284 | 1111919 | 0.000081 | 5.6 | 52 | 495 | 97820082 |
| Qh (L/hr/body) | 10000 | 93 | 0 | 93 | 93 | 93 | 93 | 93 |
| CL_h (L/hr/body) | 10000 | 27 | 33 | 0.0000023 | 0.81 | 8.5 | 50 | 93 |
| Kpuu_tumour | 10000 | 257628 | 15558580 | 1 | 1.1 | 2.6 | 56 | 1417291971 |
| CLint_tumour (L/hr/body) | 10000 | 14 | 366 | 0.0000000069 | 0.0014 | 0.019 | 0.25 | 23421 |
| CLinflux_target (L/hr/body) | 10000 | 156 | 3314 | 0.000065 | 0.18 | 1.2 | 9.3 | 249552 |
| CLint_targetMet (L/hr/body) | 10000 | 14 | 366 | 0.0000000069 | 0.0014 | 0.019 | 0.25 | 23421 |
| Clint_all_target (L/hr/body) | 10000 | 43 | 1760 | 0.000000099 | 0.005 | 0.055 | 0.55 | 167784 |
| fbClint_all_target (L/hr/body) | 10000 | 8.8 | 177 | 0.0000000021 | 0.00072 | 0.0099 | 0.12 | 10290 |
| CL_mTarget (L/hr/body) | 10000 | 0.048 | 0.067 | 0.0000000021 | 0.00072 | 0.0094 | 0.076 | 0.21 |

Supplementary Table 2: Examples of the virtual compounds’ key pharmacokinetic parameters and associated parameters used for the simulation.

| Virtual Compound ID | Vmax hepatic uptake (µmol/h) | Km hepatic uptake (µmol/L) | Vmax hepatic metabolism (µmol/h) | Km hepatic metabolism (µmol/L) | Vmax target uptake (µmol/h) | Km target uptake (µmol/L) | Vmax target metabolism (µmol/h) | Km target metabolism (µmol/L) |
| --- | --- | --- | --- | --- | --- | --- | --- | --- |
| 5 | 3.13 | 0.2610 | 7,420.000 | 210.000 | 0.7910 | 0.0908 | 0.319000 | 3.380 |
| 10 | 10,400.00 | 2.6200 | 257.000 | 33.000 | 705.0000 | 0.0401 | 0.015300 | 3.620 |
| 43 | 81.70 | 0.3510 | 8,220.000 | 2.350 | 0.0409 | 0.4420 | 0.010200 | 2.140 |
| 76 | 3.94 | 1.0000 | 17,800.000 | 6.250 | 8.2300 | 1.4000 | 0.003750 | 0.914 |
| 95 | 6,340.00 | 0.4770 | 0.102 | 5.380 | 1.4700 | 0.0442 | 0.182000 | 1.740 |
| 89 | 975.00 | 0.4370 | 122.000 | 14.200 | 14.0000 | 0.3940 | 0.002750 | 258.000 |
| 198 | 25,300.00 | 0.8800 | 599.000 | 8.510 | 0.2040 | 0.5060 | 0.017600 | 37.400 |
| 431 | 20.10 | 0.8210 | 1,460.000 | 0.916 | 0.0163 | 0.6360 | 0.117000 | 12.800 |
| 784 | 35,800.00 | 0.0682 | 10.400 | 28.000 | 6.9800 | 1.8000 | 0.344000 | 1.380 |
| Virtual Compound ID | Clint_all (L/hr) | CLh (L/hr) | CLr (L/hr) | fb (ratio) | Kd (µmol/L) | k_on (L/(µmol h)) | koff (1/h) | Receptor abundance (µmol) |
| 5 | 32.000 | 9.560 | 6.7400 | 0.33300 | 0.0011800 | 109.00 | 0.1280 | 0.000310 |
| 10 | 382.000 | 24.900 | 0.2240 | 0.08910 | 0.0013600 | 7.92 | 0.0108 | 0.027900 |
| 43 | 245.000 | 66.000 | 94.1000 | 0.92500 | 0.0000788 | 130,000.00 | 10.2000 | 0.011800 |
| 76 | 22.400 | 1.150 | 0.3900 | 0.05190 | 0.0051200 | 1,990.00 | 10.2000 | 0.445000 |
| 95 | 0.258 | 0.138 | 1.9000 | 0.53700 | 0.0098300 | 103.00 | 1.0200 | 0.000472 |
| 89 | 175.000 | 40.000 | 24.5000 | 0.40100 | 0.0448000 | 21.00 | 0.9420 | 0.000435 |
| 198 | 776.000 | 12.700 | 0.1670 | 0.01900 | 0.0000872 | 62,400.00 | 5.4400 | 0.172000 |
| 431 | 39.500 | 26.500 | 8.4900 | 0.93900 | 0.0023000 | 594.00 | 1.3600 | 0.003540 |
| 784 | 5,390.000 | 24.100 | 0.0231 | 0.00604 | 0.0000396 | 386,000.00 | 15.3000 | 0.010800 |
| Virtual Compound ID | Estimated Therapeutic dose (µmol) | ITM dose (µmol) | ITM_aveRO (%) | IV_aveRO (%) | IV_AUC24central (µmol h) |  |  |  |
| 5 | 20.0 | 0.200 | 35.4 | 63.9 | 1.2300 |  |  |  |
| 10 | 3.0 | 0.030 | 83.8 | 65.4 | 0.1190 |  |  |  |
| 43 | 20.0 | 0.200 | 92.6 | 76.7 | 0.1380 |  |  |  |
| 76 | 5.0 | 0.050 | 6.11 | 62.8 | 2.9900 |  |  |  |
| 95 | 2.0 | 0.020 | 6.29 | 70.9 | 0.8590 |  |  |  |
| 89 | 0.8 | 0.008 | 80.5 | 62.3 | 0.0125 |  |  |  |
| 198 | 50.0 | 0.250 | 38.7 | 61.9 | 3.9900 |  |  |  |
| 431 | 5.0 | 0.050 | 25.6 | 61.2 | 0.1490 |  |  |  |
| 784 | 40.0 | 0.250 | 40.5 | 62.2 | 0.9110 |  |  |  |


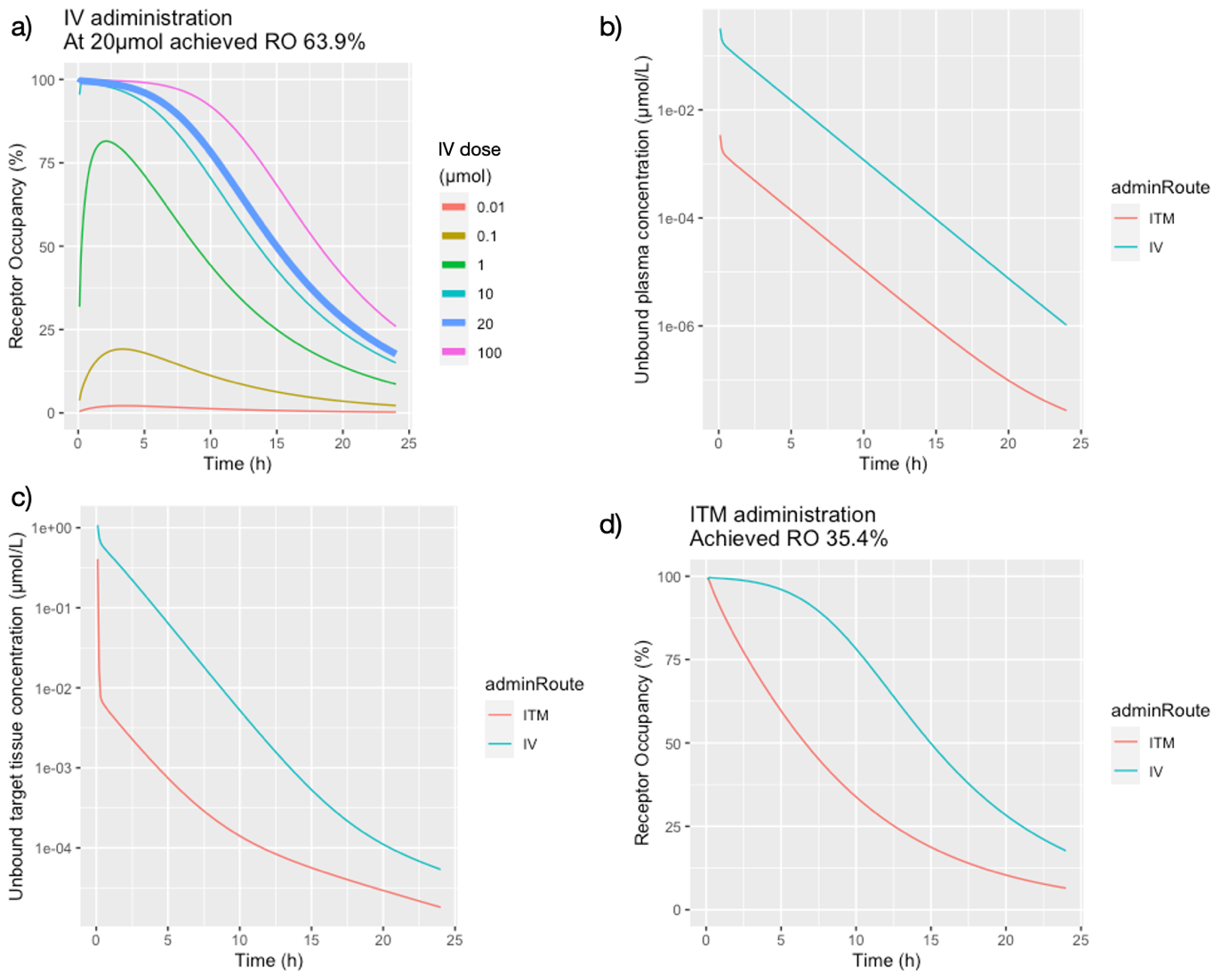


Supplementary Figure 1: Simulation outcomes for Virtual Compound 5, illustrating the time-dependent receptor occupancy and drug concentration profiles:

a) Receptor occupancy across varying intravenous (IV) doses. The thick line represents the minimum dose that achieves an average receptor occupancy (RO) exceeding 60%, identified as the estimated therapeutic dose.

b) Comparison of unbound plasma concentration profiles following IV administration at the estimated therapeutic dose and an intra-target microdose (ITM), where the microdose is calculated based on the estimated therapeutic dose.

c) Profiles of unbound drug concentration within the target tissue after IV administration at the estimated therapeutic dose and the ITM, highlighting the pharmacokinetic outcomes of each dosing approach.

d) RO profiles following IV administration at the estimated therapeutic dose versus the ITM, illustrating the efficacy of each dosing strategy.


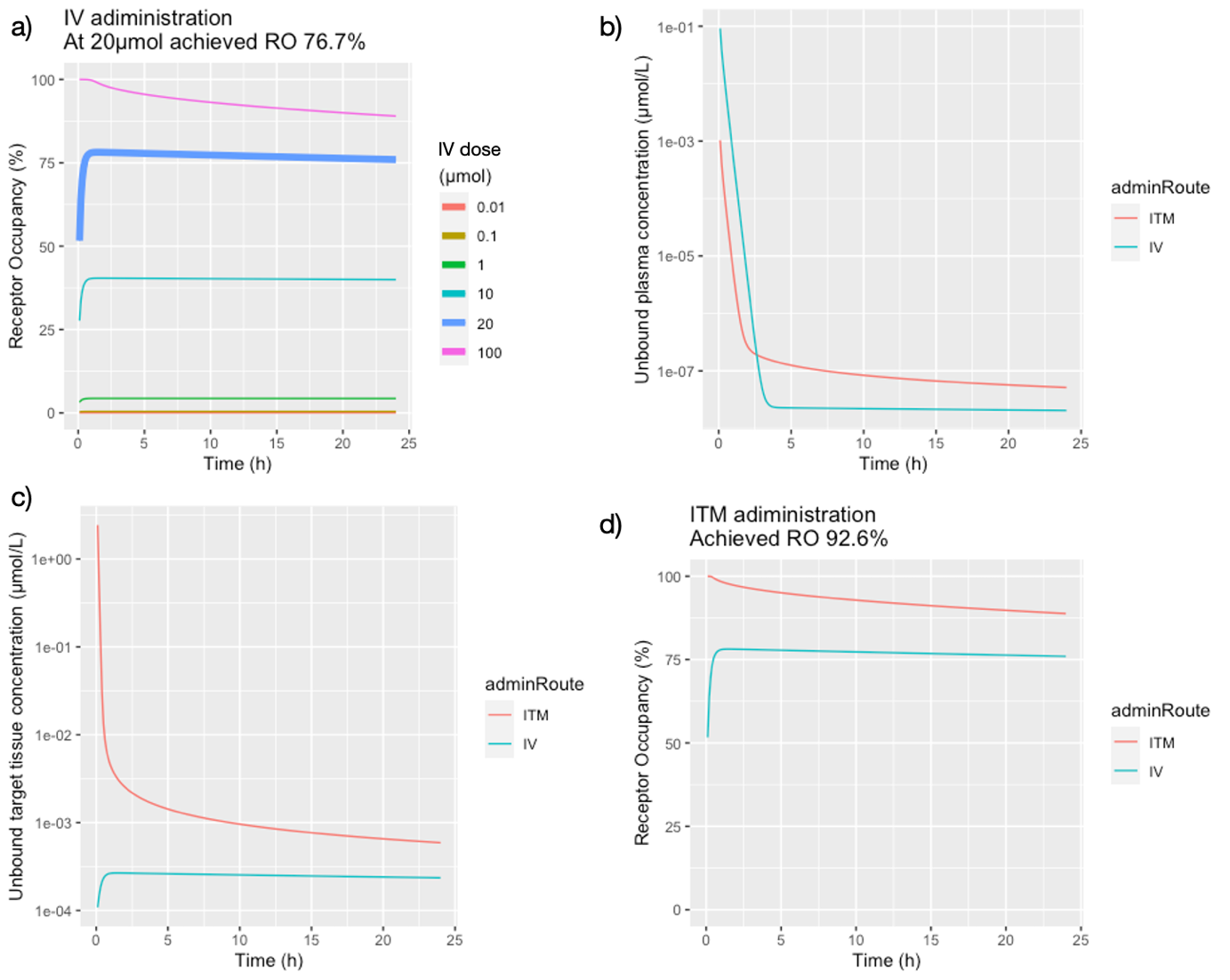


Supplementary Figure 2: Simulation outcomes for Virtual Compound 10, illustrating the time-dependent receptor occupancy and drug concentration profiles:

a) Receptor occupancy across varying intravenous (IV) doses. The thick line represents the minimum dose that achieves an average receptor occupancy (RO) exceeding 60%, identified as the estimated therapeutic dose.

b) Comparison of unbound plasma concentration profiles following IV administration at the estimated therapeutic dose and an intra-target microdose (ITM), where the microdose is calculated based on the estimated therapeutic dose.

c) Profiles of unbound drug concentration within the target tissue after IV administration at the estimated therapeutic dose and the ITM, highlighting the pharmacokinetic outcomes of each dosing approach.

d) RO profiles following IV administration at the estimated therapeutic dose versus the ITM, illustrating the efficacy of each dosing strategy.


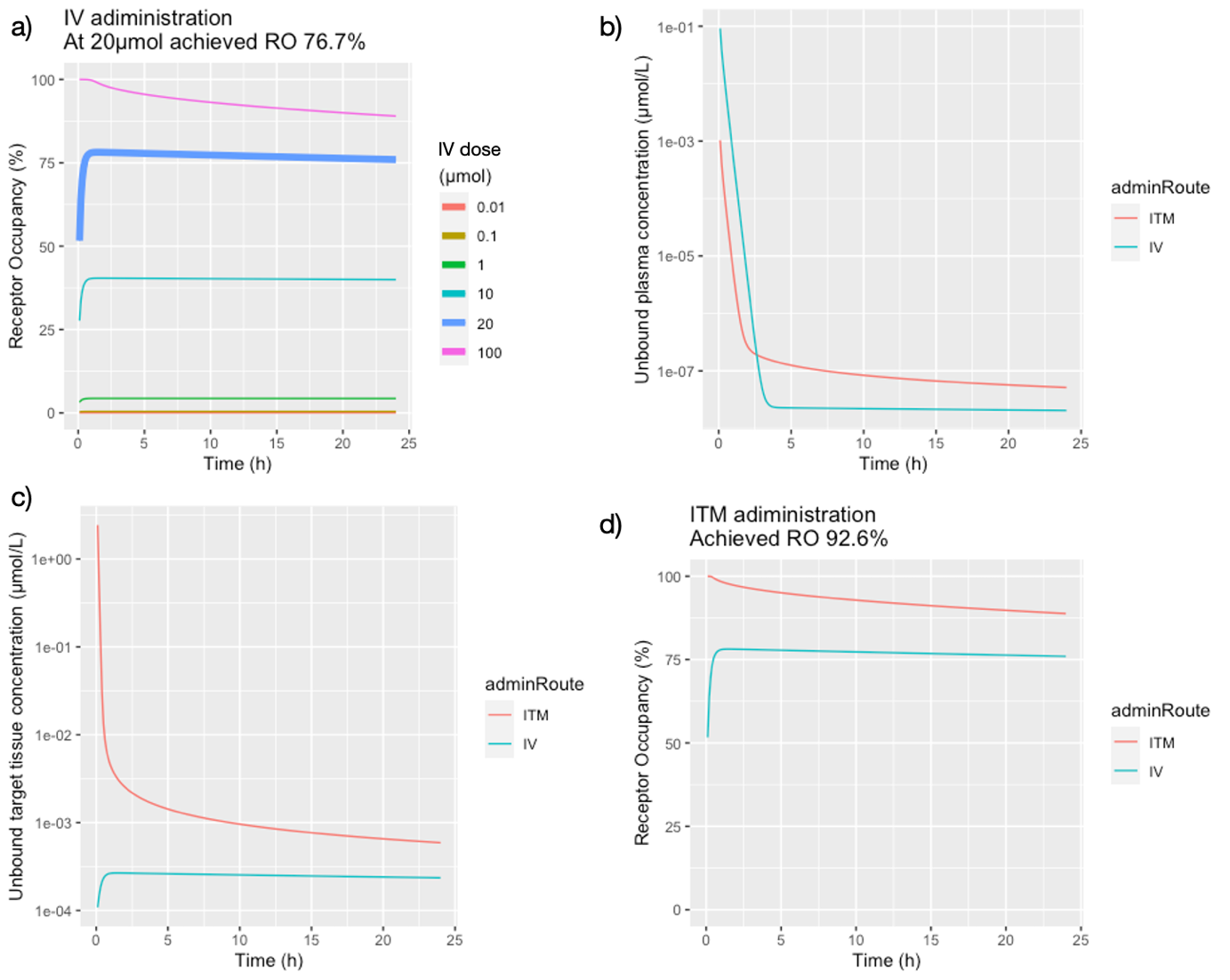


Supplementary Figure 3: Simulation outcomes for Virtual Compound 43, illustrating the time-dependent receptor occupancy and drug concentration profiles:

a) Receptor occupancy across varying intravenous (IV) doses. The thick line represents the minimum dose that achieves an average receptor occupancy (RO) exceeding 60%, identified as the estimated therapeutic dose.

b) Comparison of unbound plasma concentration profiles following IV administration at the estimated therapeutic dose and an intra-target microdose (ITM), where the microdose is calculated based on the estimated therapeutic dose.

c) Profiles of unbound drug concentration within the target tissue after IV administration at the estimated therapeutic dose and the ITM, highlighting the pharmacokinetic outcomes of each dosing approach.

d) RO profiles following IV administration at the estimated therapeutic dose versus the ITM, illustrating the efficacy of each dosing strategy.


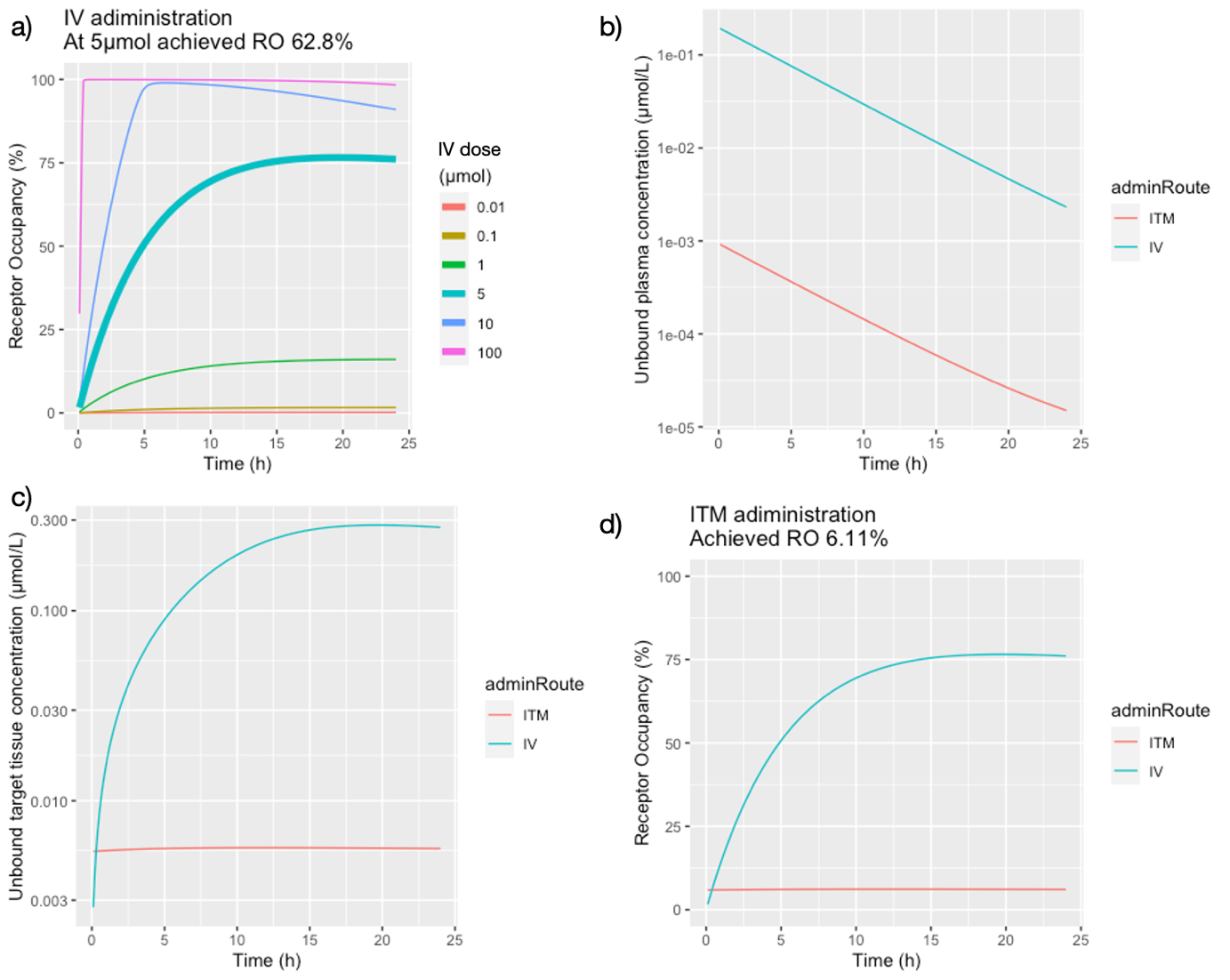


Supplementary Figure 4: Simulation outcomes for Virtual Compound 76, illustrating the time-dependent receptor occupancy and drug concentration profiles:

a) Receptor occupancy across varying intravenous (IV) doses. The thick line represents the minimum dose that achieves an average receptor occupancy (RO) exceeding 60%, identified as the estimated therapeutic dose.

b) Comparison of unbound plasma concentration profiles following IV administration at the estimated therapeutic dose and an intra-target microdose (ITM), where the microdose is calculated based on the estimated therapeutic dose.

c) Profiles of unbound drug concentration within the target tissue after IV administration at the estimated therapeutic dose and the ITM, highlighting the pharmacokinetic outcomes of each dosing approach.

d) RO profiles following IV administration at the estimated therapeutic dose versus the ITM, illustrating the efficacy of each dosing strategy.


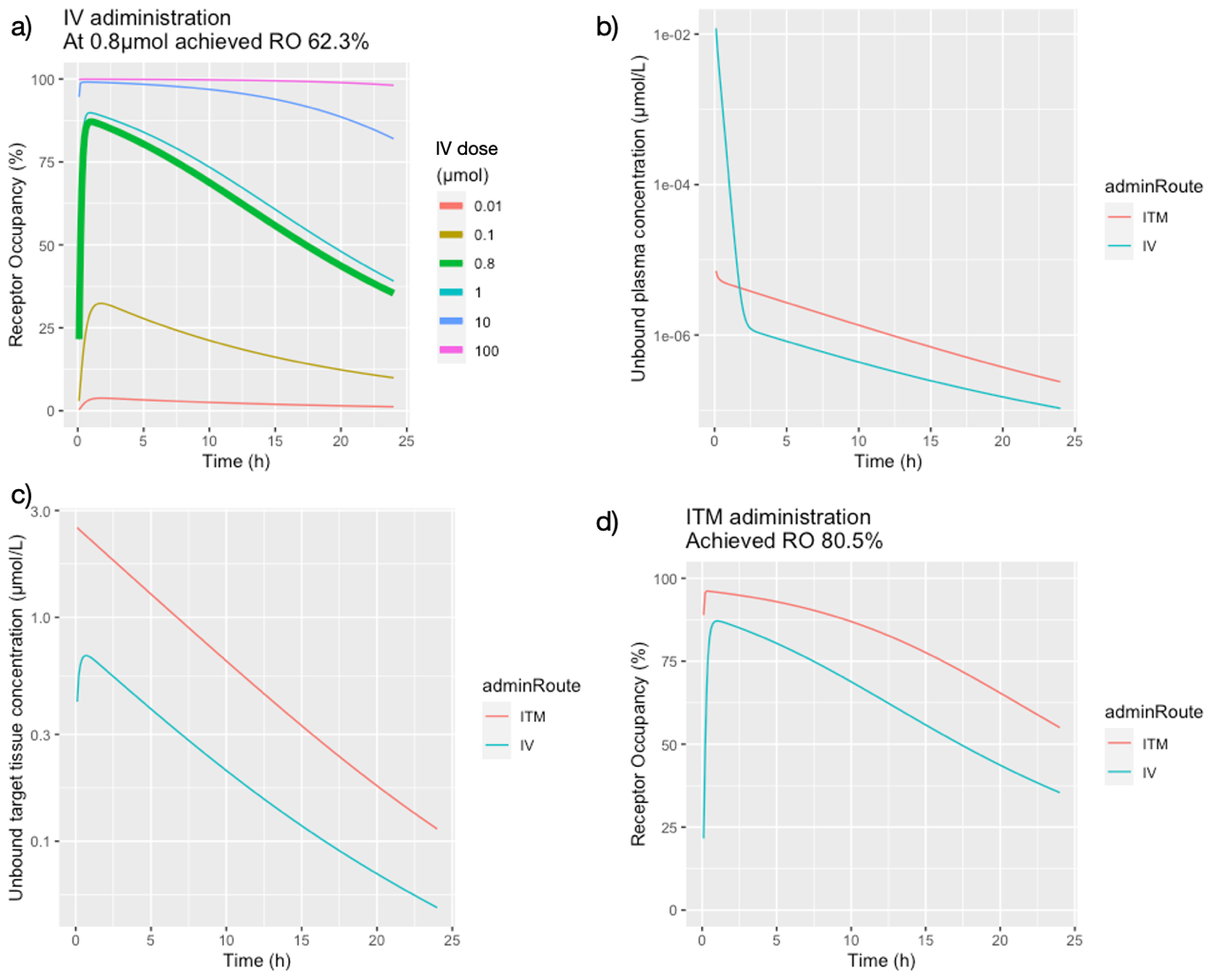


Supplementary Figure 5: Simulation outcomes for Virtual Compound 89, illustrating the time-dependent receptor occupancy and drug concentration profiles:

a) Receptor occupancy across varying intravenous (IV) doses. The thick line represents the minimum dose that achieves an average receptor occupancy (RO) exceeding 60%, identified as the estimated therapeutic dose.

b) Comparison of unbound plasma concentration profiles following IV administration at the estimated therapeutic dose and an intra-target microdose (ITM), where the microdose is calculated based on the estimated therapeutic dose.

c) Profiles of unbound drug concentration within the target tissue after IV administration at the estimated therapeutic dose and the ITM, highlighting the pharmacokinetic outcomes of each dosing approach.

d) RO profiles following IV administration at the estimated therapeutic dose versus the ITM, illustrating the efficacy of each dosing strategy.


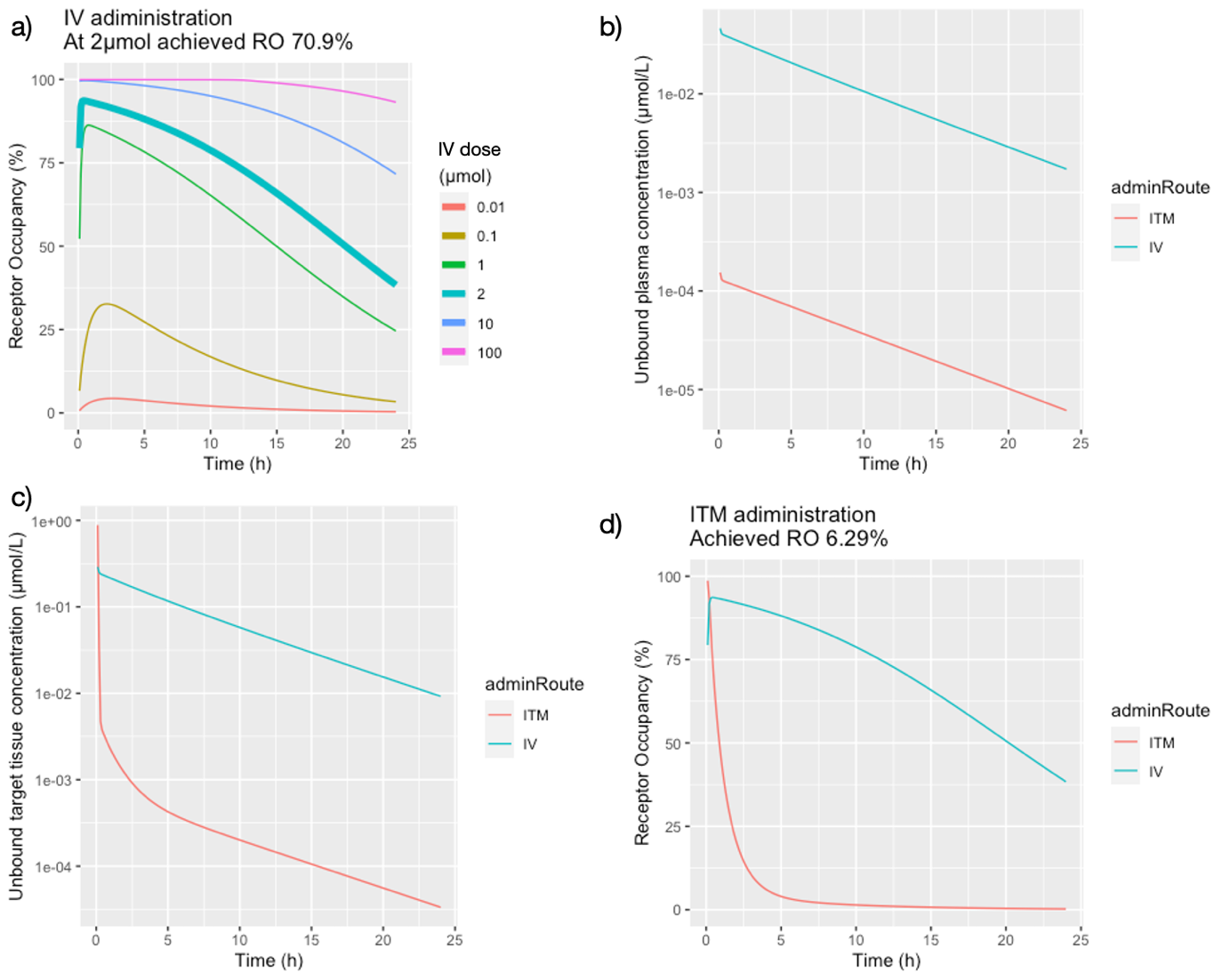


Supplementary Figure 6: Simulation outcomes for Virtual Compound 95, illustrating the time-dependent receptor occupancy and drug concentration profiles:

a) Receptor occupancy across varying intravenous (IV) doses. The thick line represents the minimum dose that achieves an average receptor occupancy (RO) exceeding 60%, identified as the estimated therapeutic dose.

b) Comparison of unbound plasma concentration profiles following IV administration at the estimated therapeutic dose and an intra-target microdose (ITM), where the microdose is calculated based on the estimated therapeutic dose.

c) Profiles of unbound drug concentration within the target tissue after IV administration at the estimated therapeutic dose and the ITM, highlighting the pharmacokinetic outcomes of each dosing approach.

d) RO profiles following IV administration at the estimated therapeutic dose versus the ITM, illustrating the efficacy of each dosing strategy.


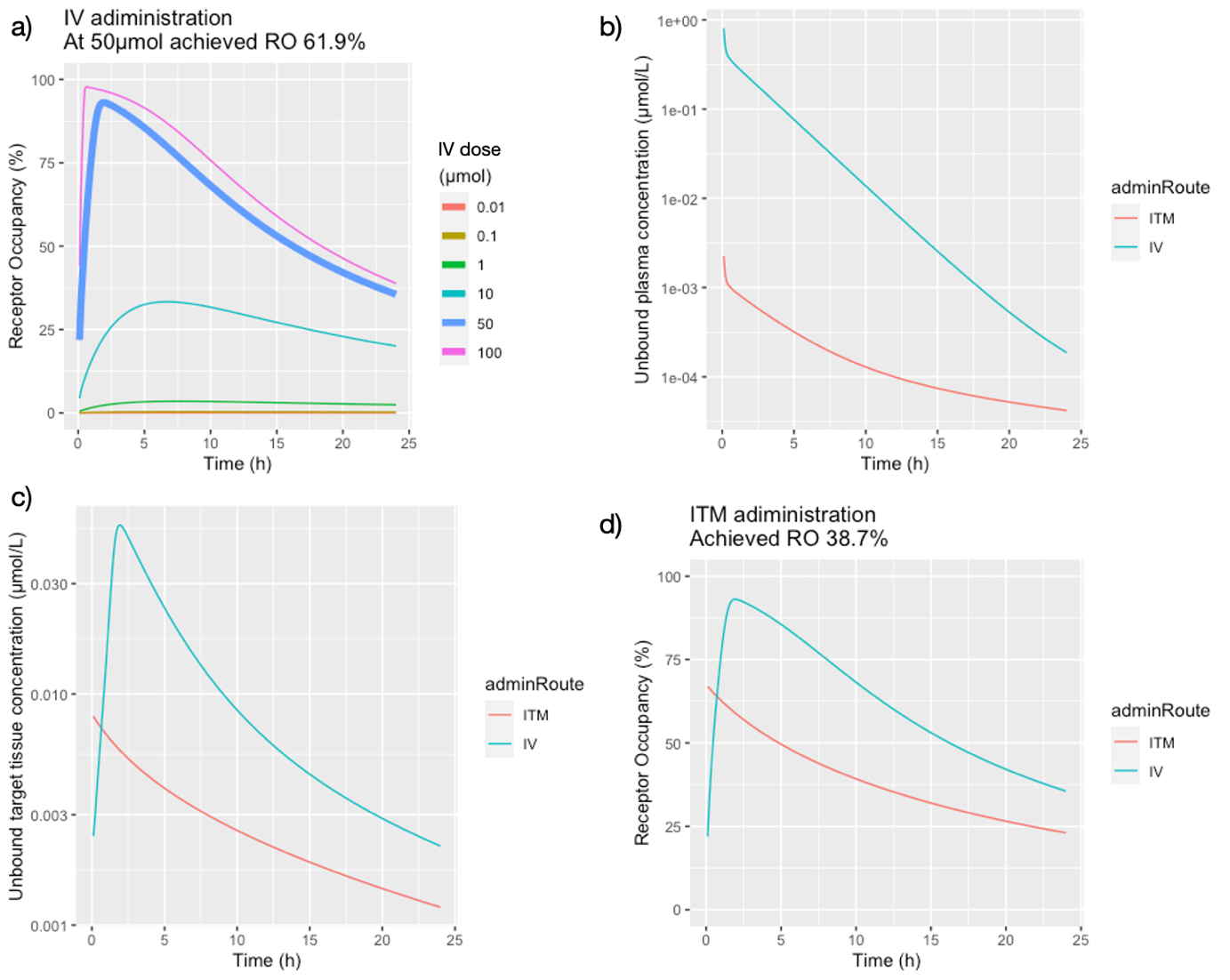


Supplementary Figure 7: Simulation outcomes for Virtual Compound 198, illustrating the time-dependent receptor occupancy and drug concentration profiles:

a) Receptor occupancy across varying intravenous (IV) doses. The thick line represents the minimum dose that achieves an average receptor occupancy (RO) exceeding 60%, identified as the estimated therapeutic dose.

b) Comparison of unbound plasma concentration profiles following IV administration at the estimated therapeutic dose and an intra-target microdose (ITM), where the microdose is calculated based on the estimated therapeutic dose.

c) Profiles of unbound drug concentration within the target tissue after IV administration at the estimated therapeutic dose and the ITM, highlighting the pharmacokinetic outcomes of each dosing approach.

d) RO profiles following IV administration at the estimated therapeutic dose versus the ITM, illustrating the efficacy of each dosing strategy.


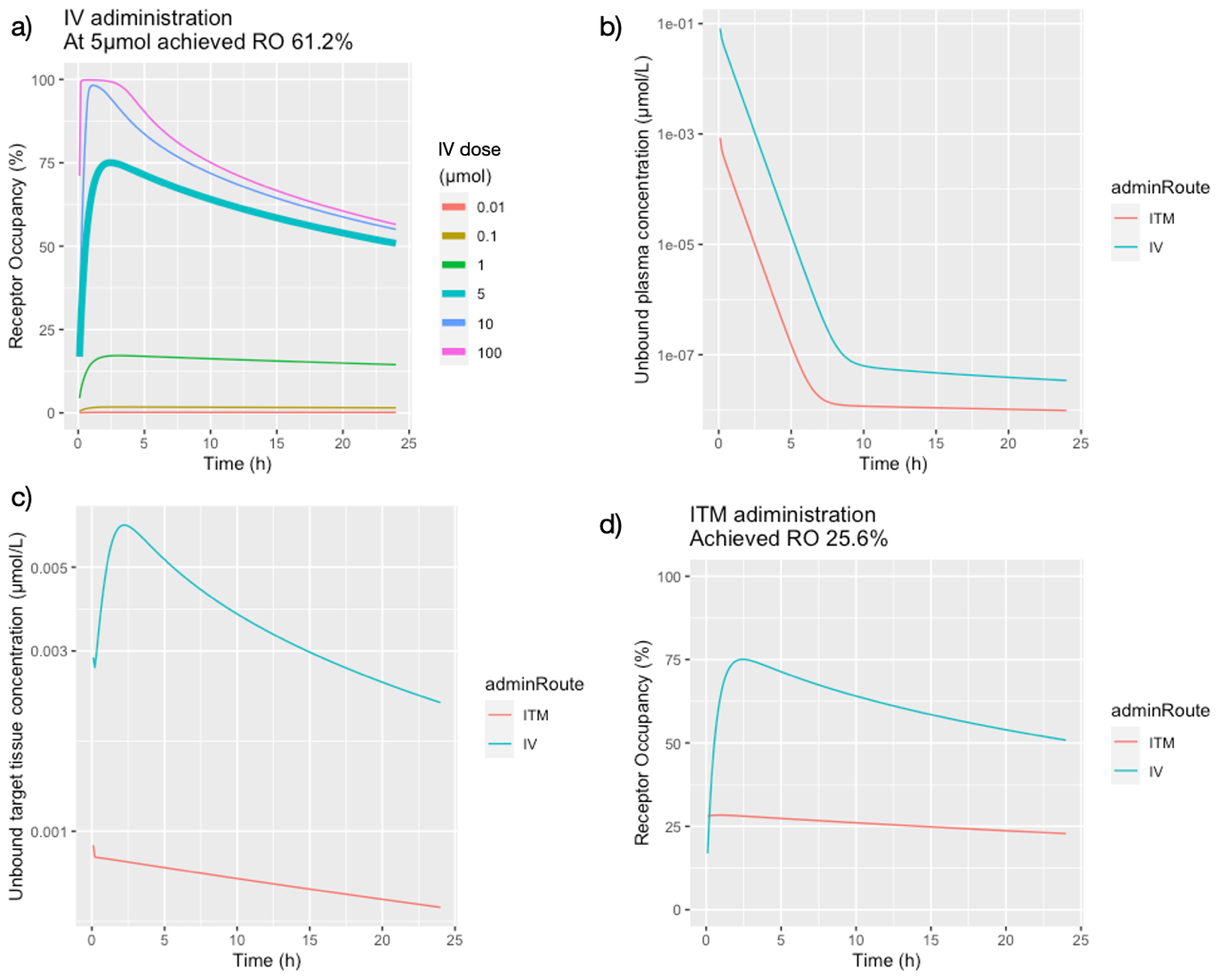


Supplementary Figure 8: Simulation outcomes for Virtual Compound 431, illustrating the time-dependent receptor occupancy and drug concentration profiles:

a) Receptor occupancy across varying intravenous (IV) doses. The thick line represents the minimum dose that achieves an average receptor occupancy (RO) exceeding 60%, identified as the estimated therapeutic dose.

b) Comparison of unbound plasma concentration profiles following IV administration at the estimated therapeutic dose and an intra-target microdose (ITM), where the microdose is calculated based on the estimated therapeutic dose.

c) Profiles of unbound drug concentration within the target tissue after IV administration at the estimated therapeutic dose and the ITM, highlighting the pharmacokinetic outcomes of each dosing approach.

d) RO profiles following IV administration at the estimated therapeutic dose versus the ITM, illustrating the efficacy of each dosing strategy.


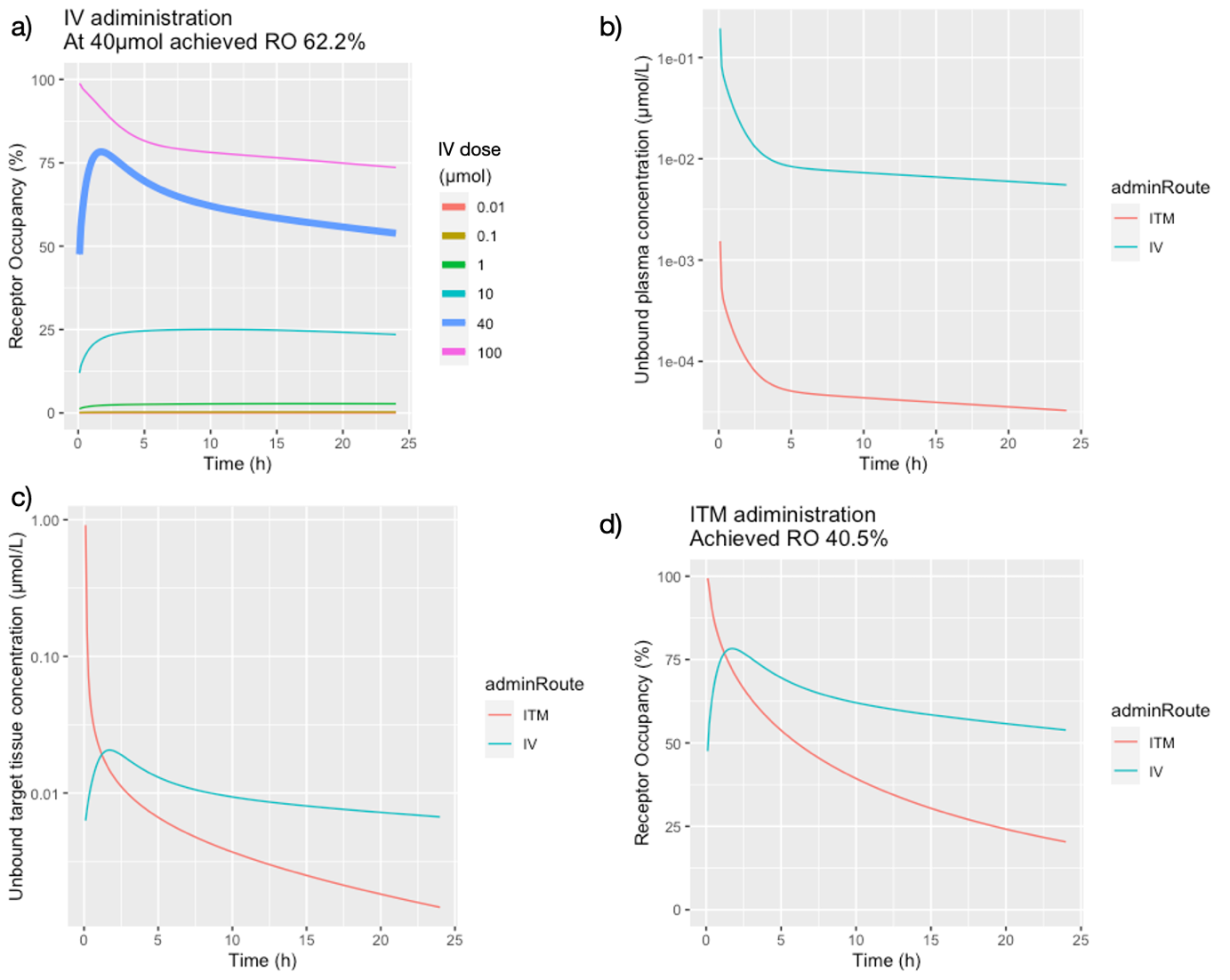


Supplementary Figure 9: Simulation outcomes for Virtual Compound 784, illustrating the time-dependent receptor occupancy and drug concentration profiles:

a) Receptor occupancy across varying intravenous (IV) doses. The thick line represents the minimum dose that achieves an average receptor occupancy (RO) exceeding 60%, identified as the estimated therapeutic dose.

b) Comparison of unbound plasma concentration profiles following IV administration at the estimated therapeutic dose and an intra-target microdose (ITM), where the microdose is calculated based on the estimated therapeutic dose.

c) Profiles of unbound drug concentration within the target tissue after IV administration at the estimated therapeutic dose and the ITM, highlighting the pharmacokinetic outcomes of each dosing approach.

d) RO profiles following IV administration at the estimated therapeutic dose versus the ITM, illustrating the efficacy of each dosing strategy.


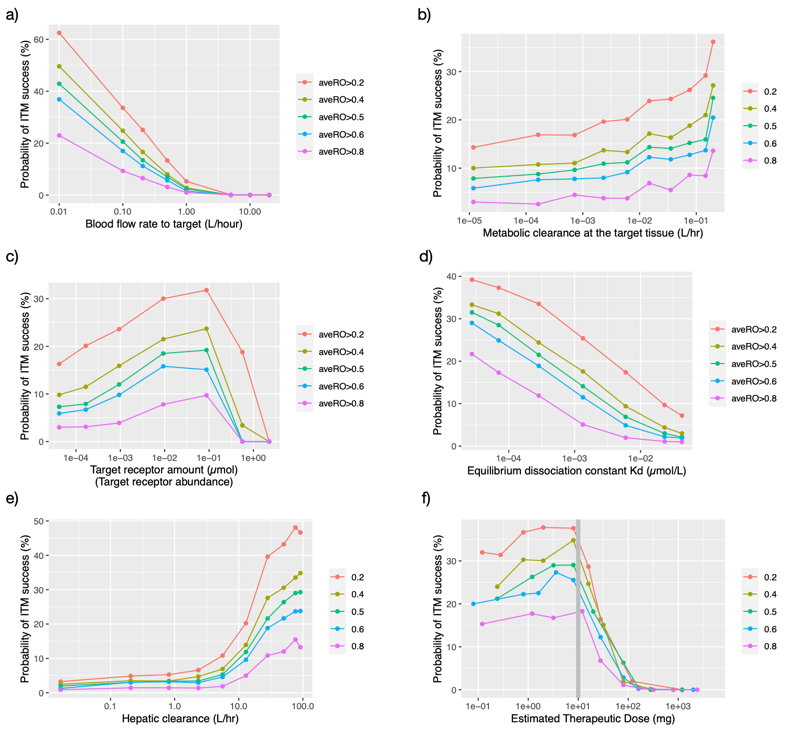


Supplementary Figure 10: Relationships between probability of ITM success and various key target organ, target receptor, and pharmacokinetic parameters. ITM success is defined as achieving an average receptor occupancy of over 20,40,50,60, or 80% during the 24-hour period following ITM administration.

a) Blood flow rate to the target tissue.

b) Metabolic clearance rate within the target tissue.

c) Target receptor abundance.

d) Equilibrium dissociation constant (Kd) for the drug-target receptor complex.

e) Hepatic clearance rate.

f) Estimated therapeutic dose.


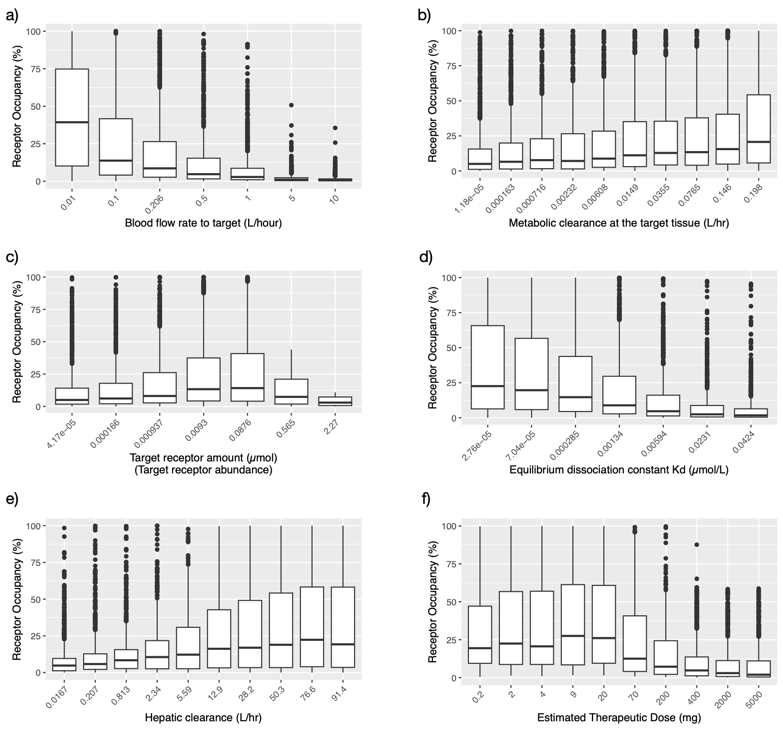


Supplementary Figure 11: Distribution of average receptor occupancy via ITM with various key target organ, target receptor, and pharmacokinetic parameters. The ITM dose was decided based on the estimated therapeutic dose achieving an average receptor occupancy of over 60% during the 24-hour period after IV administration.

a) Blood flow rate to the target tissue.

b) Metabolic clearance rate within the target tissue.

c) Target receptor abundance.

d) Equilibrium dissociation constant (Kd) for the drug-target receptor complex.

e) Hepatic clearance rate.

f) Estimated therapeutic dose.


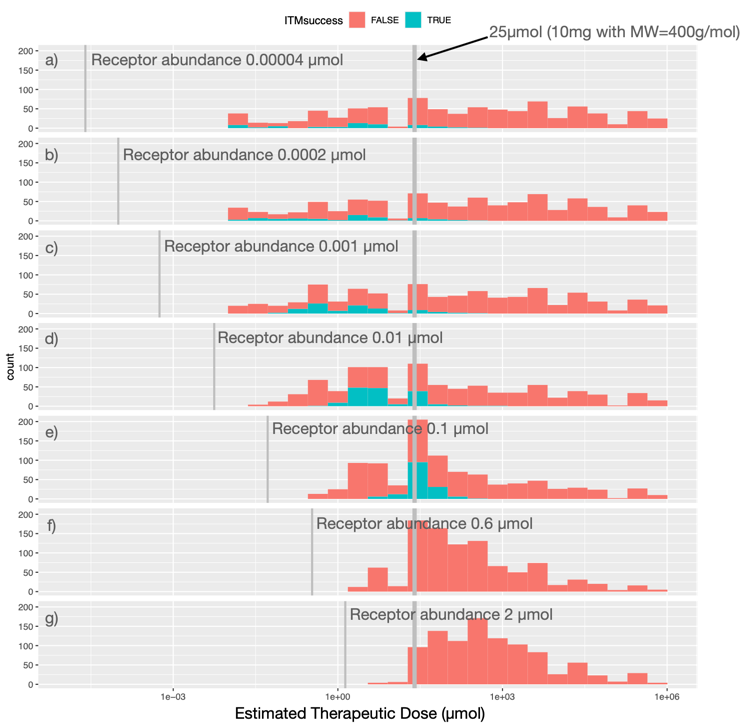


Supplementary Figure 12: Distribution of therapeutic dose with various receptor abundance. The thin vertical lines correspond to the receptor abundances, thick bold lines correspond to 25µmol which is equivalent of 10mg given the assumed molecular weight (MW) of 400g/mol.

a) when receptor abundance is 40pmol we have observed ITM success on 59 out of 1000 virtual compound.

b) when receptor abundance is 200pmol we have observed ITM success on 67 out of 1000 virtual compound.

c) when receptor abundance is 1nmol we have observed ITM success on 98 out of 1000 virtual compound.

d) when receptor abundance is 10nmol we have observed ITM success on 158 out of 1000 virtual compound.

e) when receptor abundance is 100nmol we have observed ITM success on 151 out of 1000 virtual compound.

f) when receptor abundance is 600nmol we have observed ITM success on 0 out of 1000 virtual compound.

g) when receptor abundance is 2mmol we have observed ITM success on 0 out of 1000 virtual compound.
